# Patterns of pharmacotherapy for bipolar disorder: A GBC survey

**DOI:** 10.1111/bdi.13366

**Published:** 2023-07-18

**Authors:** Balwinder Singh, Anastasia K. Yocum, Rebecca Strawbridge, Katherine E. Burdick, Caitlin E. Millett, Amy T. Peters, Sarah H. Sperry, Giovanna Fico, Eduard Vieta, Norma Verdolini, Ophelia Godin, Marion Leboyer, Bruno Etain, Ivy F. Tso, Brandon J. Coombes, Melvin G. McInnis, Andrew A. Nierenberg, Allan H. Young, Melanie M. Ashton, Michael Berk, Lana J. Williams, Kamyar Keramatian, Lakshmi N. Yatham, Bronwyn J. Overs, Janice M. Fullerton, Gloria Roberts, Philip B. Mitchell, Ole A. Andreassen, Ana C. Andreazza, Peter P. Zandi, Daniel Pham, Joanna M. Biernacka, Mark A. Frye, The FACE-BD Collaborators, The Global Bipolar Cohort Collaborative

**Affiliations:** 1 Mayo Clinic, Department of Psychiatry & Psychology, Mayo Clinic, Rochester, Minnesota, USA; 2 Department of Psychiatry, University of Michigan, Ann Arbor, Michigan, USA; 3 Department of Psychological Medicine, Institute of Psychiatry, Psychology & Neuroscience, King’s College London, London, UK; 4 Harvard Medical School, Brigham and Women’s Hospital, Boston, Massachusetts, USA; 5 Dauten Family Center for Bipolar Treatment Innovation, Harvard Medical School, Massachusetts General Hospital, Boston, Massachusetts, USA; 6 Bipolar and Depressive Disorders Unit, Institute of Neuroscience, Hospital Clinic, University of Barcelona, IDIBAPS, CIBERSAM, Barcelona, Catalonia, Spain; 7 Local Health Unit Umbria 1, Department of Mental Health, Mental Health Center of Perugia, Perugia, Italy; 8 INSERM U955, IMRB, Translational Neuro-Psychiatry, Fondation FondaMental, Univ Paris Est Créteil, Créteil, France; 9 Département Médico-Universitaire de Psychiatrie et d’Addictologie (DMU IMPACT), APHP, Hôpitaux Universitaires Henri Mondor, Fédération Hospitalo-Universitaire de Médecine de Précision en Psychiatrie (FHU ADAPT), Créteil, France; 10 Groupe Hospitalo-universitaire AP-HP Nord, DMU Neurosciences, Hôpital Fernand Widal, Département de Psychiatrie et de Médecine Addictologique, INSERM UMRS 1144, Université de Paris, AP-HP, Paris, France; 11 Department of Psychiatry & Behavioral Health, The Ohio State University, Columbus, Ohio, USA; 12 Department of Quantitative Health Sciences, Mayo Clinic, Rochester, Minnesota, USA; 13 IMPACT –the Institute for Mental and Physical Health and Clinical Translation, School of Medicine, Barwon Health, Deakin University, Geelong, Victoria, Australia; 14 Orygen, The National Centre of Excellence in Youth Mental Health, Centre for Youth Mental Health, Florey Institute for Neuroscience and Mental Health and the Department of Psychiatry, The University of Melbourne, Melbourne, Victoria, Australia; 15 Department of Psychiatry, University of British Columbia, Vancouver, Canada; 16 Neuroscience Research Australia, Randwick, Sydney, New South Wales, Australia; 17 School of Medical Sciences, Faculty of Medicine, University of New South Wales, Sydney, New South Wales, Australia; 18 School of Psychiatry, Faculty of Medicine, University of New South Wales, Sydney, New South Wales, Australia; 19 NORMENT Centre, Division of Mental Health and Addiction, University of Oslo and Oslo University Hospital, Oslo, Norway; 20 Department of Pharmacology & Toxicology, Temerty Faculty of Medicine, University of Toronto, Toronto, Canada; 21 Department of Psychiatry and Behavioral Sciences, Johns Hopkins School of Medicine, Baltimore, Maryland, USA; 22 The Milken Institute, Washington, District of Columbia, USA

**Keywords:** antidepressants, antipsychotics, bipolar disorder, lithium, mood stabilizer, pharmacotherapy

## Abstract

**Objectives:**

To understand treatment practices for bipolar disorders (BD), this study leveraged the Global Bipolar Cohort collaborative network to investigate pharmacotherapeutic treatment patterns in multiple cohorts of well-characterized individuals with BD in North America, Europe, and Australia.

**Methods:**

Data on pharmacotherapy, demographics, diagnostic subtypes, and comorbidities were provided from each participating cohort. Individual site and regional pooled proportional meta-analyses with generalized linear mixed methods were conducted to identify prescription patterns.

**Results:**

This study included 10,351 individuals from North America (*n* = 3985), Europe (*n* = 3822), and Australia (*n* = 2544). Overall, participants were predominantly female (60%) with BD-I (60%; vs. BD-II = 33%). Cross-sectionally, mood-stabilizing anticonvulsants (44%), second-generation antipsychotics (42%), and antidepressants (38%) were the most prescribed medications. Lithium was prescribed in 29% of patients, primarily in the Australian (31%) and European (36%) cohorts. First-generation antipsychotics were prescribed in 24% of the European versus 1% in the North American cohort. Antidepressant prescription rates were higher in BD-II (47%) compared to BD-I (35%). Major limitations were significant differences among cohorts based on inclusion/exclusion criteria, data source, and time/year of enrollment into cohort.

**Conclusions:**

Mood-stabilizing anticonvulsants, second-generation antipsychotics, and antidepressants were the most prescribed medications suggesting prescription patterns that are not necessarily guideline concordant. Significant differences exist in the prescription practices across different geographic regions, especially the underutilization of lithium in the North American cohorts and the higher utilization of first-generation antipsychotics in the European cohorts. There is a need to conduct future longitudinal studies to further explore these differences and their impact on outcomes, and to inform and implement evidence-based guidelines to help improve treatment practices in BD.

## 1 | INTRODUCTION

Bipolar disorder (BD) is a chronic illness with recurrent mood episodes/symptoms that can lead to significant functional impairment.^1,2^ Best practices for the management of BD often focus on mood stabilizers for long-term stabilization.^3,4^ The bipolar pharmacopoeia has evolved gradually over the last 25 years. Several studies have explored the pharmacotherapeutic prescription practices in BD and reported variable patterns of pharmacologic treatment,^5–8^ most notably the underutilization of lithium (Li) in the United States.^9^ Based on regulatory drug development, the use of second-generation antipsychotics (SGAs) has steadily increased.^10^ Unimodal antidepressants (ADs) are not supported by regulatory approval and guideline recommendations, but are prescribed in 40%–50% of BD patients despite limited long-term evidence and guidelines often proscribing use.^9,10^ Multimodal pharmacotherapy and multiple antipsychotic prescriptions are increasing, leading to significant drug interactions and higher side effect burdens.^11^

Prior studies have highlighted differential prescriptions of mood stabilizing anticonvulsants (MSACs) and Li (underutilization), and first-generation antipsychotics (FGAs; higher prescription) in BD patients of African ancestry versus European ancestry.^12–14^ A recent study investigating the prescription patterns for BD in Asian countries reported that >80% of patients received mood stabilizers (MSs) and/or antipsychotics, with 20% of patients receiving complex polypharmacy.^7^ An international survey investigating pharmacotherapeutic practices across different geographic regions would help to understand the global variabilities in BD treatment and potential outcomes.

The Global Bipolar Cohort (GBC) is an international alliance of research institutions with existing longitudinal and cross-sectional studies in several countries across the world. Researchers in the GBC, who have been convening formally since 2019, have now established collaborations and are forming a partnership to investigate studies of individuals with BD. The mission of the GBC is to identify current best practices globally to improve treatment outcomes for patients with BD, and continually promote collaboration among its institutional members.^15^ The GBC does not represent all ongoing BD research worldwide, but rather represents a starting point and an outline of an iterative process.^1^ In a recent article, the GBC identified high rates of functional impairment (41%–75%) in patients with BD across 13 studies from seven countries.^1^ The GBC provides a unique opportunity to leverage these independent studies to identify both consistencies and differences in prescription practices for well-characterized BD patients from around the world.

In this study, we aimed to investigate patterns of pharmacologic treatments across several sites in North America, Europe, and Australia, leveraging the GBC collaborative network. We hypothesized that there would be differences in prescription rates across different geographic regions, especially for Li, SGAs, FGAs, and ADs.

## 2 | METHODS

A cross-sectional survey was sent to the research investigators who are part of the GBC initiative. Participants with Bipolar I Disorder (BD-I), Bipolar II Disorder (BD-II), schizoaffective disorder-bipolar type (SCZ-BD), and BD not otherwise specified (BD-NOS) were included. To minimize diagnostic heterogeneity, SCZ-BD and BD-NOS data were not included in the BD diagnostic subtype analysis. Eleven independent sites across North America, Europe, and Australia provided aggregated, cohort-level data. Each site was asked to provide detailed characteristics of their cohort including demographics, diagnostic/clinical subtypes, and comorbidities at the time of enrollment or study initiation. Inclusion criteria included DSM-IV diagnosis, proficiency in English, euthymic, or affective stability for a varying time before enrollment, ability to provide informed consent, and greater than 18 years of age. Exclusion criteria varied but most studies excluded participants who were actively psychotic, or suicidal, with current substance abuse or neurologic disease including dementia, had an IQ score <85, or were pregnant or breastfeeding. Data were gathered either by structured interview, self-report, or electronic health records between 1998 and 2020. We requested prevalence data in aggregate regarding medication use (Current use Yes/No; and if yes, mean dose, if available).

To compare demographic variables including sex, race, diagnosis, body mass index (BMI), history of psychotic symptoms, history of psychotic symptoms with mania, comorbid substance use disorders (SUD), and comorbid anxiety disorders across geographic variables, tests of equal proportions, two-sided without continuity correction, were used. Proportional meta-analysis was conducted using generalized linear mixed methods (GLMM), a random intercept logistic regression model. Pharmaceutical classes were compared using the logit transformation summary method of PLOGIT and between-study τ^2^ based on the maximum-likelihood estimator (ML). We primarily focused on pharmacopeia for BD-I and BD-II only—namely Li, MSACs (valproate, carbamazepine, and lamotrigine), SGAs, FGAs, ADs with/without antimanic mood stabilizers, stimulants/wakefulness agents (modafinil, armodafinil) with/without antimanic MSs, sedative-hypnotics (benzodiazepines, Z-drugs) with/without antimanic MSs, dopamine agonists, and patients not taking any medications. We investigated differences across regions (North America vs. Europe vs. Australia) and independently across the individual sites, stratified by diagnostic subtypes. If a site did not provide diagnostic subtype data (BD-I and BD-II), we did not include its data in the diagnostic subtype analysis. All statistical analysis was completed in R version 4.2.0 (2022–04-22; Vigorous Calisthenics) using dplyr (1.0.9), meta (5.5–0), metasens (1.5–0), gemtc (1.0–1), rjags (4–13), ggplot2 (3.3.6), and stats (4.2.0).

## 3 | RESULTS

A total of 10,351 individuals from 11 independent studies from North America (*n* = 3985), Europe (*n* = 3822), and Australia (*n* = 2544) were included. Individual study descriptions and inclusion/exclusion criteria are presented by site in Table S1a,b, respectively. All sites provided cross-sectional data, except for the NeuRA cohort,^16,17^ which reported medication supply from government administrative records over 14 years (File S2). Furthermore, as bipolar subtypes derived from ICD-10-AM diagnosis codes are unreliable, the NeuRA cohort was excluded from diagnostic subtype analyses. Overall, rates of bipolar subtypes were BD-I 60%, BD-II 33%, BD-NOS 5%, and 2% with SCZ-BD (Table 1) and were statistically different across regions. Rates of comorbid anxiety disorders and history of SUD, not stratified by diagnosis, were significantly higher in the North American sites (62% and 55%, respectively) compared with European (37% for both anxiety and SUD) and Australian sites (30% and 14%, respectively). Rates of lifetime psychosis were higher in the Australian sites, while lifetime mania with psychotic features was more prevalent in North American sites.

The most prescribed pharmaceutical classes for all regions and all diagnoses combined were MSACs (44%), SGAs (42%), and ADs (38%) (Figure S1 and Table S1c). Cross-sectionally, Li was prescribed to 29% of patients, averaged across all regions, and was least prescribed in North American (23%) compared to Australian (31%) and European (36%) sites. Li was more commonly prescribed in men compared to women (32% vs. 28%), whereas the rates of MSACs and SGAs were similar among men and women (Figure S2). Antidepressant prescription rates were higher in women compared to men (40% vs. 34%) using the common-effect model. Figure S1(a–l) shows the meta-analysis for the overall prescription rates cross-sectionally, irrespective of diagnosis subtypes, for Li, MSACs, SGAs, FGAs, ADs, benzodiazepines, non-benzodiazepine sedatives, two or more SGAs, two or more MSs, three or more MSs, no MSs, and no medications, presented by each site. Figure S3(a–l) shows the same, but includes the NeuRA data, which had notably higher rates of AD use (79%) over a 14-year period. On average, 18% of the patients were not on any psychotropic medications or on any MS (Li/MSAC/SGA). Polypharmacotherapy (two or more MSs) was reported in 32% of the cross-sectional sample, 3% and 8% were on two or more ADs or SGAs, respectively. FGAs were prescribed in 24% of patients in the European sites compared to only 1% of patients in the North American sites and 2% in the Australian sites. Two or more SGAs were prescribed more frequently in the European sites (14%) compared to the Australian (5%) and North American (3%) sites. These results show significant proportional differences as indicated by a low *p*-value (*p* < 0.01); however, high heterogeneity measures such as *I*^2^ and τ^2^ indicate a large observed variance and high dispersion of true effect sizes.

### 3.1 | Bipolar disorders

**FIGURE 1:**
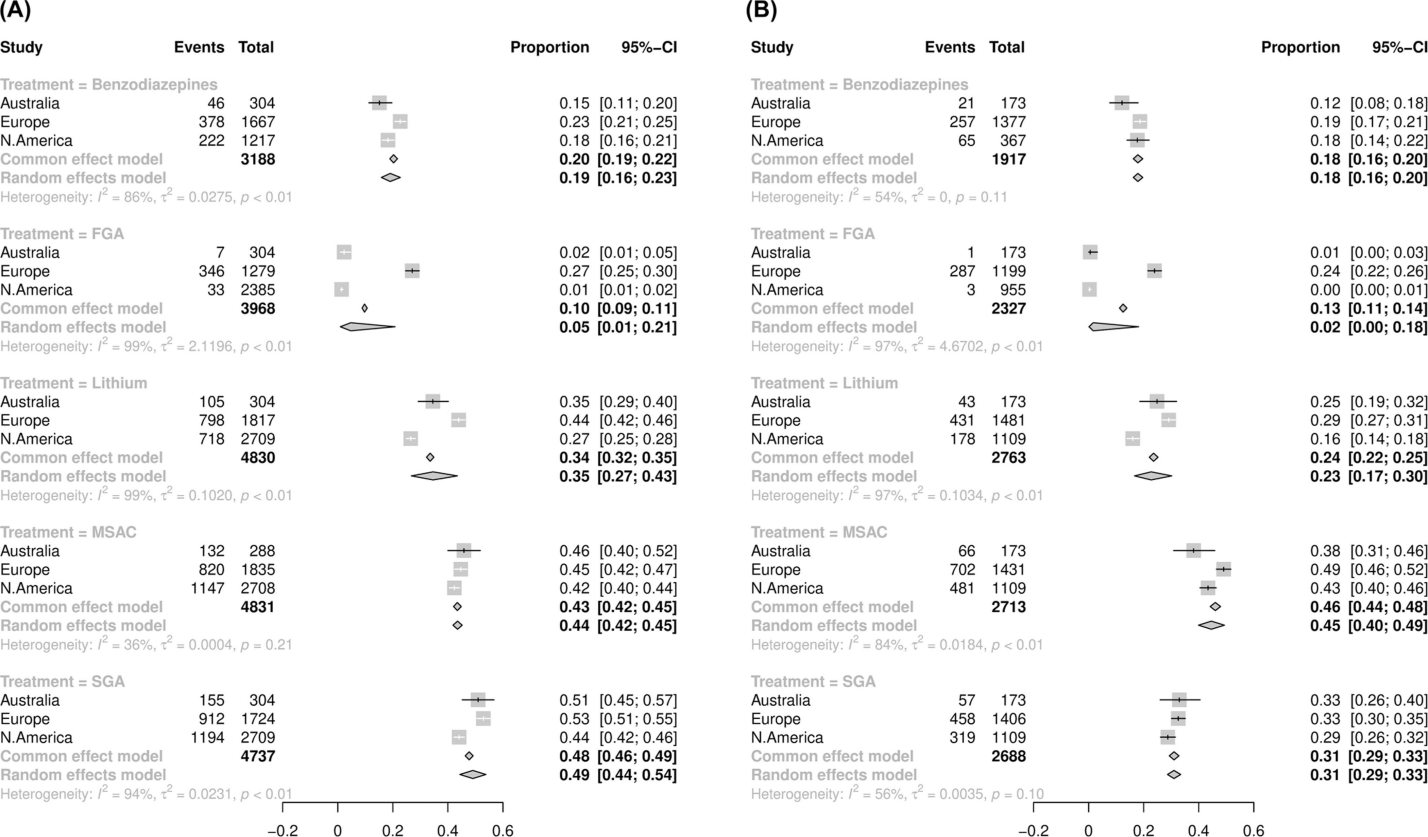
Proportional meta-analysis (GLMM) for the common pharmacopeia in bipolar disorder across three geographical regions, stratified by diagnosis, (A) bipolar-I disorder and (B) bipolar-II disorder.

Figure 1 shows patterns of pharmacologic treatments (benzodiazepines, FGAs, Li, SGAs, and MSACs) based on diagnostic subtypes (BD-I and BD-II) across the regions. There was significant variability in prescription practices. FGAs were most prescribed for BD-I and BD-II in the European sites compared to those in North America and Australia (*p* < 0.01). The rates of Li prescriptions in BD-I and BD-II were significantly lower in the North American sites (27% and 16%, respectively) compared to European (44% and 29%, respectively) and Australian sites (35% and 25%, respectively). The MSACs were prescribed similarly across regions for BD-I (*p* = 0.21, *I*^2^ = 36%, τ^2^ = 0.0004), whereas, for BD-II, we observed a higher prescription rate of MSACs in the European sites (49%) compared to the Australian (38%) and North American (43%) sites (*p* < 0.01). In BD-I, the prescription rates for SGAs were higher in European sites (53%) compared to the North American (44%) and Australian (51%) sites. In BD-II, the prescription rates for SGAs were similar across regions (*p* = 0.10, *I*^2^ = 56%, τ^2^ = 0.0035). Benzodiazepines were prescribed significantly more in the European compared to the Australian and North American sites for BD-I, but were not differently prescribed across regions in BD-II (*p* = 0.11, *I*^2^ = 54%, τ^2^ = 0).

**FIGURE 2:**
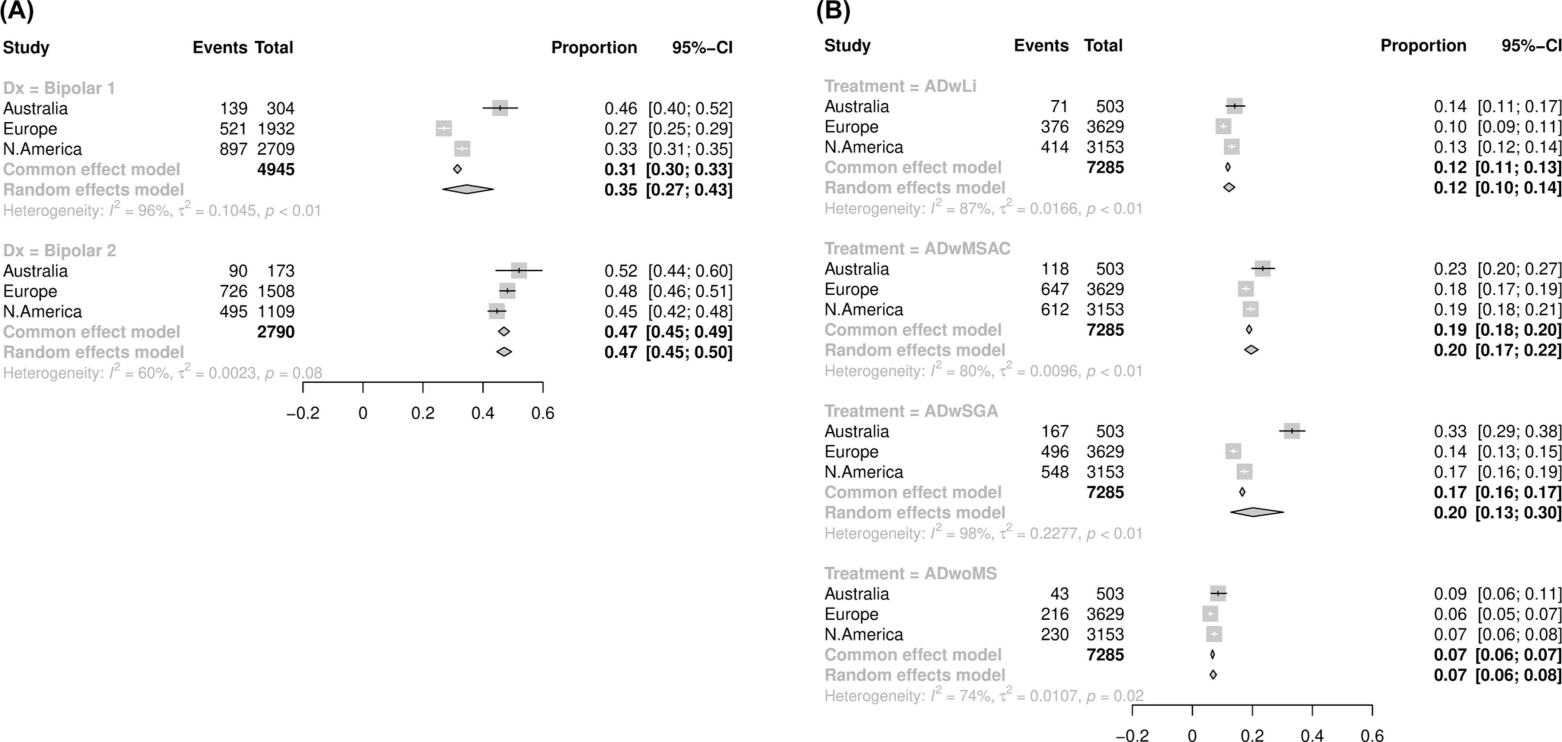
Proportional meta-analysis (GLMM) for antidepressants pooled by region stratified by diagnosis (A) and antidepressants in combination with or without other psychotropics (B). The NeuRA cohort was excluded from this summary due to its longitudinal ascertainment of medication use. The corresponding figure including the NeuRA data can be found in Figure S4.

Figure 2A shows variable patterns of AD prescriptions based on diagnostic subtypes (BD-I and BD-II) across regions. Antidepressant prescription rates, on average, across regions were higher in BD-II (47%) compared to BD-I (35%). Prescription rates for ADs in BD-I were significantly higher (*p* < 0.01) for the Australian sites (46%) compared to European (27%) and North American sites (33%). However, the rates of AD prescription were similar across regions for BD-II (45%–52%; *p* = 0.08, *I*^2^ = 60%, τ^2^ = 0.0023). Combination therapies with ADs were significantly different across regions without stratifying by diagnostic subtype; they were particularly common in the Australian sites in comparison to European and North American sites, shown in Figure 2B. Further, three regions showed significantly different prescribing of ADs without MSs; (6%–9%; *p* = 0.02, *I*^2^ = 74%, τ^2^ = 0.0107), the highest in the Australian sites, Figure 2B. The proportion of SUD history compared with the rate of AD use showed a nonsignificant correlation across nine sites, *R*^2^ = 0.15 (*p* = 0.29). However, there was a stronger correlation for comorbid anxiety with proportions using ADs across 10 sites, *R*^2^ = 0.32 (*p* = 0.11). Figure S4(a–c) shows the proportional meta-analysis (GLMM) for ADs pooled by region including the NeuRA cohort.

**FIGURE 3:**
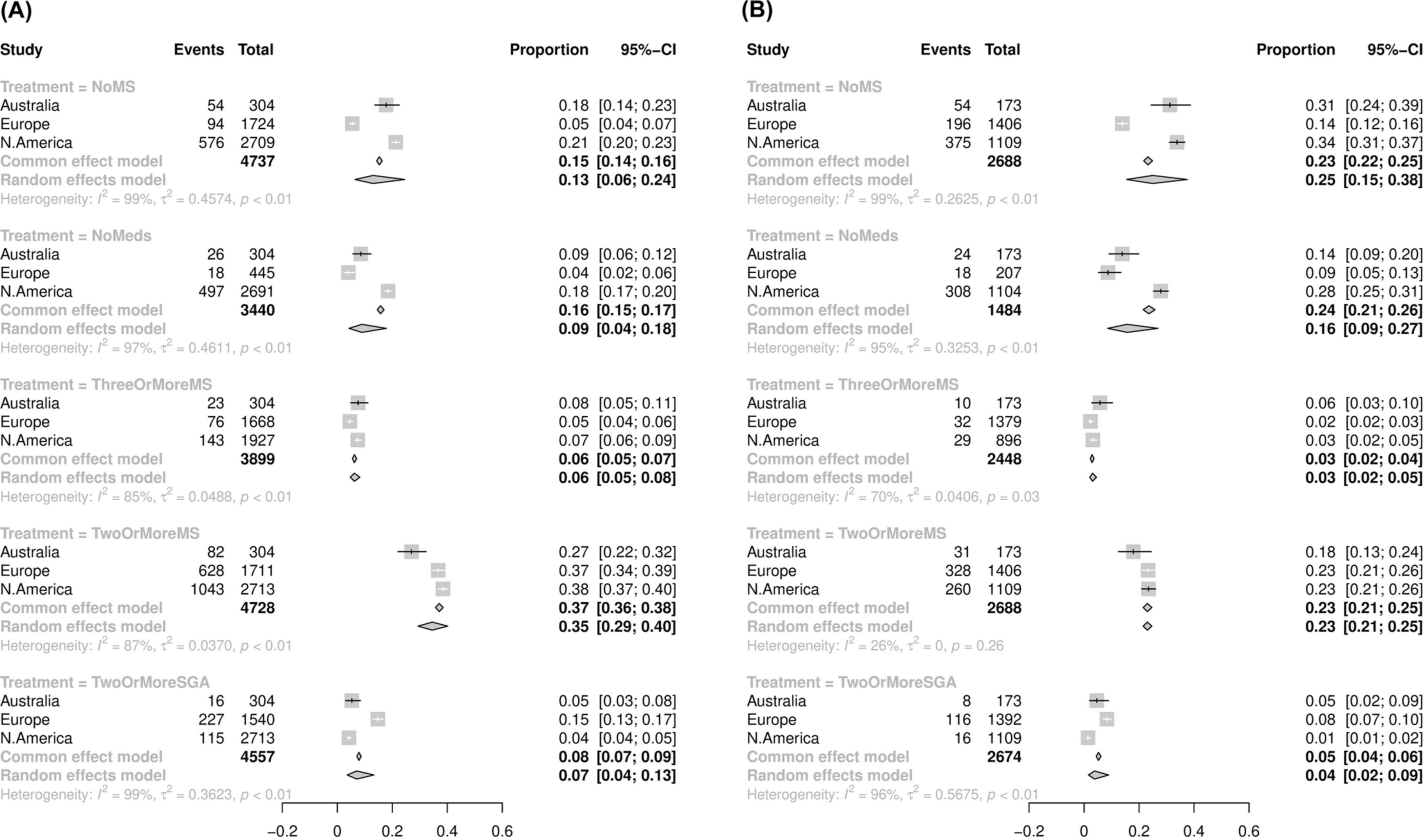
Proportional meta-analysis (GLMM) for absent or combination psychotropic prescription use pooled by region stratified by diagnosis; Bipolar-I (A) and bipolar-II (B).

Figure 3 shows patterns of polypharmacotherapy (≥2 MSs) along with no medications or no MSs based on diagnostic subtypes across regions. In both BD-I (Figure 3A) and BD-II (Figure 3B), the highest proportion of patients not on any psychotropic medications or MSs were from the North American sites. European sites had significantly fewer participants not on any psychotropic medication regardless of diagnosis. The rates of two or more SGA prescriptions for BD-I were significantly higher in the European sites (15%) compared to the Australian or North American sites (5% or 4%, respectively). However, Australian sites were found to prescribe the greatest proportion of three or more MSs independently of diagnosis (8% for Australian vs. 5% and 7% for European and North American sites, respectively). Overall, the average number of psychotropics per participant across all cross-sectional sites was 2 with a range of 1.08–2.35, whereas the prevalence of multiple psychotropics in the NeuRA sample averaged 5.25 (standard deviation = 3.28) over 14 years.

Race-related data were not available in some of the European and Australian sites but for those available, race was primarily Caucasian. As such, we were not able to conduct a priori planned meta-analysis across regions to explore race-based variable patterns of pharmacologic treatments. Medication dose data were not provided by all the sites and thus was not able to be analyzed.

## 4 | DISCUSSION

This multisite survey characterized patterns of pharmacologic treatments across North America, Europe, and Australia. Despite heterogenous data, MSACs, SGAs, and ADs were the most common medications prescribed in the collected, aggregated sample. SGAs (49%) and ADs (47%) were the most commonly prescribed medications in BD-I and BD-II, respectively. Over the last 20 years, drug development in BD, outside of lamotrigine, has focused on SGAs, potentially explaining this finding.^18^ ADs were the third-most prescribed treatment overall, with higher prescription rates in BD-II. This is consistent with recent results from a large National Ambulatory Medical Care Survey in the United States.^10^ In contrast to SGAs, prescription rates of ADs have been steadily increasing in BD, despite limited evidence for long-term use and potential risk of mood destabilization.^19–22^ Despite higher rates of anxiety disorders, there was no significant difference in ADs or benzodiazepine prescription rates geographically in BD-II. Most patients were prescribed ADs in conjunction with either a SGA (20%), MSAC (20%), or Li (12%). The risks and benefits of adjunctive AD use may have treatment implications as ADs, when added to SGAs rather than MSACs, remains a controversial and uncertain prescription practice.^23–26^ It is encouraging to note that only 7% of BD patients were on ADs without any MSs as this group (AD without MS) would be at a much higher risk of mood instability. This number is much smaller than that reported in a recent survey,^10^ and may reflect the fact that most of these studies were conducted at academic centers. Of note, we did not request separate AD without MS data for BD-I and BD-II. It is possible that more patients with BD-II were on ADs without a MS.^19^ This survey cannot comment on treatment outcomes for these prescribing practices, as analyses focused primarily on cross-sectional data. However, future longitudinal analyses could help explore outcomes of differential treatment strategies observed in this global survey.

There were significant differences in the comorbid conditions across the regions, with higher rates of obesity, anxiety disorders, and SUDs in the North American sites. Although it is beyond the scope of this study to identify the reasons for a higher frequency of SUDs, prior studies have shown similar findings with higher SUDs among patients with BD in the United States. Those with BD in the United States have an earlier age of onset and increased sensitization to stressors and substance use.^8^ There is a higher frequency of SUDs in North America in general, which could have also contributed to higher rates.^27^ Our findings are consistent with prior literature reporting higher anxiety disorder rates among patients with BD in the United States.^8^ However, despite the higher rates of anxiety disorders, we did not observe a concomitant increase in ADs or benzodiazepine prescription rates in the North American sites, compared to the European and Australian sites. This may reflect an already high prescription rate of ADs across regions.

Significant variability exists in the prescription practices across different regions, especially underutilization of Li in the North American sites and higher utilization of FGAs in the European sites. These differences could be due to a different mean age of prescribers and clinical experience between the different centers. A recent Italian study demonstrated the preferential prescription of Li versus valproate for the maintenance treatment of BD among Italian early career psychiatrists.^28^ Prescription rates for Li continue to remain relatively low in North America despite a significant evidence base favoring Li’s superior efficacy and capacity to protect against neuroprogression, suicide, and all-cause mortality.^29–38^ Low prescription rates for Li in the North American sites is consistent with prior literature.^9,10^ This could be due to heterogeneity within BD diagnoses. Prior studies suggested that less than one third of patients treated with Li monotherapy experience long-term response.^39^ It is possible that some of the patients had an inadequate response to Li or had failed Li therapy before enrolling in the included studies; this may help to explain our observed low prescription rates of Li. Insurance coverage is an issue in the United States; regular monitoring for Li, thyroid, and renal indices requires high copays and deductibles. The potential development of renal insufficiency^40,41^ and thyroid dysfunction^42^ are major concerns for Li nonprescription and even discontinuation by prescribers in the United States.^43^ It is additionally possible that preference has been given to more recently approved treatments, such as SGAs and MSACs, and/or a perception of greater difficulty in using Li from the standpoint of laboratory drug monitoring and drug toxicity profile. We could not pursue race-based analyses due to the Australian and European collections containing largely only Caucasian individuals. This remains an area of active investigation, as there are significant differences in the prescription practices based on race and ethnicity, according to previous research.^12,44,45^

The higher prescription of FGAs across the European sites is supported by another recent study from Europe (Italy) where 30% of BD-I patients were prescribed FGAs.^46^ The FondaMental Advanced Centers of Expertise for Bipolar Disorders (FACE-BD) cohort^47^ had the highest FGA prescription rate (25%) compared to the other sites (0%–10%). Lower FGA prescription rates in the North American sites are consistent with most evidence-based guidelines for BD.^3^ However, the seminal Clinical Antipsychotic Trials of Intervention Effectiveness (CATIE) study highlighted that perphenazine (a FGA) is equally as effective as some of the SGAs in treatment of schizophrenia.^48^ The evidence-base for FGA, as a class for acute mania is clear, but not for bipolar depression; a higher prescription of FGA could reflect a difference in health-care systems and highlight an important difference in clinical practice across regions.

We also observed a high prevalence of polypharmacotherapy use across sites. This is consistent with prior studies reporting an increasing trend of polypharmacotherapy in BD.^11,49^ However, this may reflect an enrichment of treatment-refractory patients in many sites (such as Mayo Clinic, MGH, University of Michigan, and others) that are specialty, tertiary referral centers. Polypharmacotherapy is not only widely practiced but also the standard of care in treatment-resistant cases, and is typical to many other chronic disorders such as heart diseases and cancers.^50^ It could also reflect the limited efficacy of contemporary treatments for many patients with BD.

There were significant differences among cohorts based on inclusion/exclusion criteria, data source (electronic health record, research case report form, and pharmacy claims database), cross-sectional/longitudinal medication exposure data, and time of enrollment into respective study sites (from 1998 to 2022), all of which could have impacted the observed differential prescription patterns. The AD prescription rates in patients with BD-I were higher in the Australian sites than in the other areas. However, a cross-sectional survey cannot provide information regarding causality. Our survey did not request medical comorbidities such as chronic pain, migraine, fibromyalgia, and the like. If the Australian sites had a higher rate of these comorbidities, that could contribute to a higher prescription of ADs. Another reason could be that prescribers feel more comfortable prescribing ADs in the Australian sites for BD. Prominent Australian researchers have proposed SSRIs as MSs^51^ and this could also be reflected in clinical practice. These intriguing findings need to be investigated in prospective studies. We cannot infer that this sample is representative of whole populations or geographic regions, as most data were obtained from tertiary referral centers which may bias toward treatment refractory or complex cases. However, most patients reported medication data at the time of enrollment, thus our findings may reflect community practices rather than the prescription practices at the included tertiary referral sites of data collection. We did not have data on all relevant factors that could help to explain heterogeneity, such as rapid cycling, economic status, or other comorbid diagnoses that may influence clinicians’ choice of pharmacologic treatment. One site (NeuRA) reported data derived from medicine supply aggregated over 14 years of administrative health records in a community-based sample, which may not be representative of wider populations,^52^ or those attending tertiary referral centers. Furthermore, as hospital diagnostic codes cannot reliably partition clinical subtypes, the large NeuRA dataset was not able to provide diagnostic subtype data for subgroup analysis and was not included in the aggregate analysis. However, the other two Australian sites (Sydney and Deakin) were similar to other sites included in the study and are likely to broadly reflect prescription practices in the Australian region. Moreover, we were not able to systematically gather data on treatment doses, which are also highly relevant, especially for some specific compounds.^53^ Finally, this survey focused on cross-sectional data, and thus could not meaningfully evaluate the effects of specific prescribing patterns on functional or clinical outcomes. Future longitudinal studies are necessary to address the impact of different prescribing practices, comorbid conditions, and other dimensional clinical or psychosocial features on the patient’s treatment outcomes.^15^

In this cross-sectional survey, MSACs, SGAs, and ADs were the most prescribed medications across all sites. Further, multimodal pharmacotherapy is a common practice in patients with BD. Significant differences exist in the prescription practices across different geographic regions, especially underutilization of Li in the North American sites and higher utilization of FGAs in the European sites. In general, treatment patterns diverged significantly from the evidence-base and clinical recommendations, in particular, high rates of AD use, benzodiazepine usage, and low rates of MS use, especially Li, were observed. There is a global imperative to ensure that clinical care becomes more aligned with the evidence-base to improve outcomes. There is also an urgent need to conduct longitudinal studies to explore these differences in detail, and to develop and implement standardized treatments for BD to help improve treatment outcomes. This study, conducted using datasets from GBC members, represents a critical foundation for better understanding the current successes and limitations in the field that will help inform the development of a prospective longitudinal cohort to continue exploring the impact of pharmacologic treatments on outcomes of patients with BD.

## Supplementary Material

Supinfo

## Figures and Tables

**TABLE 1:** Demographic and clinical characteristics across the different geographic regions.^a^ Test of equal proportions was conducted to determine significant differences across the three regions.

Variables	North American	European	Australian	Total	*p*-value
Overall *N*	3985	3822	2544	10,351	
Mean age, years	39.25	42.58	49.97	10,339	<0.001
Sex, *n*	3980	3822	2544	10,346	
Female, *n* (%)	2437 (61.2%)	2300 (60.2%)	1486 (58.4%)	60.1%	0.076
Race, *n*	3960	861	2199	6871	
Caucasian, *n* (%)	3184 (80.4%)	852 (99.0%)	2050 (93.2%)	88.6%	<0.001
Non-Caucasian, *n* (%)	775 (19.6%)	9 (1%)	149 (6.8%)		
BMI, *n*	3587	3031	2474	9092	
Healthy (18.5–24.9), *n* (%)	982 (27.4%)	1493(49.3%)	726 (29.3%)	35.2%	<0.001
Overweight (25–29.9), *n* (%)	1091 (30.4%)	941 (31.0%)	829 (33.5%)	31.5%	0.076
Obese (≥30), *n* (%)	1479 (41.2%)	597 (19.7%)	756 (30.6%)	31.2%	<0.001
Diagnosis, *n*	3983	3830	503	8316	
Bipolar-I, *n* (%)	2713 (68.1%)	1941 (50.7%)	304 (60.4%)	59.6%	<0.001
Bipolar-II, *n* (%)	1109 (27.8%)	1508 (39.4%)	173 (34.4%)	33.5%	<0.001
SCZ-BD, *n* (%)	84 (2.1%)	77 (2%)	10 (2%)	2.1%	0.992
BD-NOS, *n* (%)	77 (1.9%)	304 (7.9%)	16 (3.2%)	4.7%	
History of psychotic symptoms, *n*	3880	3538	342	7760	
Psychotic symptoms, *n* (%)	1591 (41.0%)	1456 (41.2%)	186 (54.4%)	41.7%	<0.001
Manic psychotic symptoms, *n*	3398	3742	342	7482	
Manic psychotic symptoms, *n* (%)	1059 (31.2%)	1089 (29.1%)	79 (23.1%)	30.6%	0.006
Comorbid substance dx, *n*	3980	3742	2520	10,242	
SUD, *n* (%)	2184 (54.9%)	1403 (37.4%)	353 (14%)	38.5%	<0.001
Comorbid anxiety dx, *n*	3980	3811	2516	10,307	
Anxiety, *n* (%)	2464 (62%)	1410 (37%)	763 (30.3%)	45.0%	<0.001

Abbreviations: BD-NOS, Bipolar disorder not otherwise specified; BMI, Body mass index; SCZ-BD, schizoaffective disorder bipolar type; SUD, substance use disorder.

a *n* with complete data varied across different variables. Cell values that are greater than 0 but less than 5 are presented as <5.

## Data Availability

Deidentified data that underlie the results reported in this article will be made available upon reasonable request to the PI at the relevant organizing institution.
