# Supplementary material for: Patterns of pharmacotherapy for bipolar disorder: A GBC survey
Source: Bipolar Disord. Author manuscript; Available in PMC 2025 Feb 1. (PMC10794603; doi:10.1111/bdi.13366)
Supplement: Supinfo [file NIHMS1915469-supplement-Supinfo.docx]

**Patterns of Pharmacotherapy for Bipolar Disorder: A GBC Survey**

**Supplementary Material**

Table S1a. Complete Demographic and clinical characteristics of the individual cohorts

| **Geographic Regions** | **North American** | **North American** | **North American** | **North American** | **North American** | **European** | **European** | **European** | **Australian** | **Australian** | **Australian** |
| --- | --- | --- | --- | --- | --- | --- | --- | --- | --- | --- | --- |
| **Variables** | **MAYO*** | **UMICH** | **BWH/ISMMS** | **MGH** | **UBC** | **FACE-BD** | **CRiB** | **BARCELONA** | **DEAKIN** | **SYDNEY** | **NeuRA** |
| **Total N** | 2286 | 846 | 264 | 482 | 107 | 2957 | 80 | 785 | 161 | 342 | 2041 |
| **Age (years)** | 41.7 (14.9) | 49.3 (1) | 44.06 (12.1) | 38.88 (12.1) | 22.3 (4.57) | 41.0 (13.0) | 42 (18) | 44.74 (14.1) | 47.9 (12.6) | 39.82 (13.20) | 60.12 (10.1) |
| **Sex, n (%)** | 2286 | 843 | 262 | 482 | 107 | 2957 | 80 | 785 | 161 | 342 | 2041 |
| Male | 869 (38.0%) | 297 (35.2%) | 129 (49.2 %) | 199 (41.3%) | 49 (45.8%) | 1142 (38.6%) | 23 (29%) | 357 (45.5%) | 39 (24.2%) | 139 (40.6%) | 880 (43.1%) |
| Female | 1417 (62.0%) | 546 (64.5 %) | 133 (50.8 %) | 283 (58.7%) | 58 (54.2%) | 1815 (61.4%) | 57 (71%) | 428 (54.5%) | 122 (75.8%) | 203 (59.4%) | 1161 (56.9%) |
| **Race, n (%)** | 2265 | 846 | 260 | 482 | 107 | 0 | 76 | 785 | 158 | 0 | 2041 |
| Caucasian | 1897 (83.8%) | 724 (85.6%) | 129 (49.2%) | 354 (73.4%) | 80 (74.7%) | NA | 67 | 785 (100%) | 155 (96.3%) | NA | 1895 (92.8%) |
| Non-Caucasian | 367 (16.2%) | 122 (14.4%) | 131 (50%) | 128 (26.5%) | 27 (25.2%) | NA | 9 | 0 (0%) | <5 | NA | 146 (7.2%) |
| Missing | 21 | 0 | <5 | 0 | 0 | 2957 | <5 | 0 | <5 | 342 | 0 |
| **Hispanic or Latino, n (%)** | 2201 | 846 | 262 | 479 | 107 | NA | NA | NA | 158 | NA | NA |
| No | 1930 (87.7%) | 797 (94.2 %) | 209 (79.8%) | 426 (88.4%) | 107 (100%) | NA | NA | NA | 156 (98.7%) | NA | NA |
| Yes | 271 (12.3%) | 49 (5.8%) | 53 (20.2%) | 53 (11.6%) | 0.0% | NA | NA | NA | <5 | NA | NA |
| **BMI, n (%)** | 2156 | 846 | 0 | 478 | 107 | 2957 | 74 | 0 | 157 | 276 | 2041 |
| Underweight (<18.5) | 24 (1.1%) | 11 (1.3%) | NA | 0 | 0 | 0 | 0 | NA | 0 | 0 | 163 (8.0%) |
| Healthy Weight (18.5 - 24.x) | 537 (23.5%) | 245 (29.0%) | NA | 132 (27.4%) | 68 (63.5%) | 1470 (49.7%) | 23 (30%) | NA | 37 (23.6%) | 94 (27.4%) | 595 (29.2%) |
| Overweight (25-29) | 683 (29.9%) | 242 (28.6%) | NA | 134 (27.8%) | 32 (29.8%) | 911 (30.8%) | 30 (39%) | NA | 59 (37.6%) | 92 (27.1%) | 678 (33.2%) |
| Obesity (≥30) | 912 (39.9%) | 348 (41.1%) | NA | 212 (44.8%) | 7 (6.7%) | 576 (19.6%) | 21 (28%) | NA | 61 (38.9%) | 90 (26.2%) | 605 (29.6%) |
| **Diagnosis, n (%)** | 2286 | 846 | 262 | 482 | 107 | 2957 | 80 | 785 | 161 | 342 | 1250 |
| Bipolar-I | 1492 (65.3%) | 574 (67.8%) | 212 (80.9%) | 328 (68%) | 100.00% | 1376 (46.5%) | 53 (66.2%) | 503 (64.1%) | 90 (55.9%) | 214 (62.6%) | 1138 (55.8%) |
| Bipolar-II | 742 (32.8%) | 163 (19.3%) | 50 (19.1%) | 154 (32%) | NA | 1276 (43.2%) | 27 (33.8%) | 205 (26.1%) | 59 (36.7%) | 114 (33.3%) |  |
| Bipolar, NOS | NA | 77 (9.1%) | NA | NA | NA | 304 (10.3%) | NA | NA | 12 (7.5%) | <5 (1.2%) |  |
| SCZ-BD | 52 (2.3%) | 32 (3.8%) | NA | NA | NA | NA | NA | 77 (9.8%) | NA | 10 (2.9%) | 112 (5.5%) |
| **History of Psychosis, n (%)** | 2252 | 777 | 262 | 482 | 107 | 2673 | 80 | 785 | NA | 342 | NA |
| No | 1365 (60.6%) | 45.3% | 148 (56.5%) | 365 (75.7%) | 28 (26.3%) | 1638 (55.4%) | 34 (42.5%) | 410 (52.2%) | NA | 156 (45.6%) | NA |
| Yes | 887 (39.4%) | 54.7% | 114 (43.5%) | 117 (24.3%) | 79 (73.8%) | 1035 (35%) | 46 (57.5%) | 375 (47.8%) | NA | 186 (54%) | NA |
| **Manic Psychosis, n (%)** | 2252 | 777 | 262 | NA | 107 | 2957 | NA | 785 | NA | 342 | NA |
| No | 1545 (68.6%) | 570 (75.5%) | 195 (74%) | NA | 29 (27.3%) | 2093 (70.8%) | NA | 560 (71.4%) | NA | 263 (76.9%) | NA |
| Yes | 707 (31.4%) | 207 (24.5%) | 67 (26%) | NA | 78 (72.7%) | 864 (29.2%) | NA | 225 (28.6%) | NA | 79 (23.1%) | NA |
| **Current Depression Severity** | NA | 9.39 (6.7) | 7.75 (6.5) | 37.54 (14.0) | 7.5 | 10.5 (9.1) | 3.8 (2.6) | 3.8 (2.9) | 6.2 | 16.8 | NA |
| Instrument | NA | PHQ-9 | HDRS | BISS | HDRS | MADRS | HDRS | HRDS | BDRS | MADRS |  |
| **Current Mania Severity** | NA | 2.56 (3.2) | 2.71(3.5) | 19.09 (12.2) | 3.01 | 2.3 (3.5) | 2.3 (2.4) | 1.4 (1.8) | 2.8 | 5.3 | NA |
| Instrument | NA | ASRM | YMRS | BISS | YMRS | YMRS | YMRS | YMRS | YMRS | YMRS | NA |
| **Total number of Major Depressive Episodes** |  |  |  |  |  |  |  |  |  |  |  |
| N | 712 | 797 | 261 | 459 | 107 | 2957 | 80 | 785 | 161 | 174 | NA |
| Mean (SD) | 49.7 (67.4) | 24.4 (5) | 14.10 (17.5) | 39.46 (41.3) | 1.5 (2.7) | 4.2 (5.2) | 12 (14) | 7.12 (9.7) | 5.6 (6.3) | 25.83 (67.6) |  |
| **Total number of Manic episodes including current** |  |  |  |  |  |  |  |  |  |  |  |
| N | 482 | 777 | 261 | 453 | 107 | 2957 | 80 | 785 | 161 | 205 | NA |
| Mean (SD) | 27 (40.7) | 7.54 (20) | 7.9 (9.8) | 37.69 (47.5) | 1 | 5.2 (5.2) | 8 (7) | 2.72 (4.3) | 7.3 (8.7) | 17.97 (31.3) |  |
| **Comorbid substance dx, n** | 2286 | 846 | 259 | 482 | 107 | 2957 | NA | 785 | 160 | 319 | 2041 |
| No, n (%) | 1020 (44.6%) | 387 (45.7%) | 148 (57.1%) | 182 (37.8%) | 59 (54.9%) | 1904 (64.4%) | NA | 435 (55.4%) | 141 (88.1%) | 187 (58.62%) | 1839 (90.1%) |
| Yes, n (%) | 1266 (55.4%) | 459 (54.3%) | 111 (42.9%) | 300 (62.2%) | 48 (45.01%) | 1053 (35.6%) | NA | 350 (44.5%) | 19 (11.9%) | 132 (41.38%) | 202 (9.9%) |
| **Comorbid anxiety dx, n** | 2286 | 846 | 259 | 482 | 107 | 2957 | 69 | 785 | 156 | 319 | 2041 |
| No, n (%) | 764 (33.4%) | 331 (39.1%) | 122 (67.8%) | 205 (42.5%) | 94 (88.24%) | 1603 (54.2%) | 46 (67%) | 752 (95.8%) | 93 (59.6%) | 124 (39%) | 1536 (75.3%) |
| Yes, n (%) | 1522 (66.6%) | 515 (60.9%) | 137 (32.2%) | 277 (57.5%) | 13 (11.76%) | 1354 (45.8%) | 23 (33%) | 33 (4.3%) | 63 (40.4%) | 195 (61%) | 505 (24.7%) |
| Cell values that are greater than zero but less than 5 are presented as <5.  *Mayo sites has Minnesota=1240, Wisconsin=13, Florida=25, Ohio=782, Mexico=151, and Chile=75. Chile is western South America, and they represent 1.88% of the group categorized predominantly as North America.  Barcelona = University of Barcelona; BWH/ISMMS= Brigham and Women's Hospital/Icahn School of Medicine; FACE-BD = FondaMental Advanced Centers of Expertise for Bipolar Disorders; MGH = Massachusetts General Hospital; UBC= University of British Columbia; UMICH= University of Michigan; MAYO= Mayo Clinic; NeuRA = Neuroscience Research Australia | | | | | | | | | | | |

Table S1b. Inclusion, exclusion criteria along with individual study descriptives for each site.

| **Cohort** | **Inclusion criteria** | **Exclusion criteria** | **When was the date range of data collection?** | **How many patients do you have data for this measure?** | **Did you exclude patients with active suicidality/ psychosis?** | **Data source** | **When was the medication data collected?** |
| --- | --- | --- | --- | --- | --- | --- | --- |
| **MAYO** | 1) DSM-IV dx of BD-I, BD-II, or SCZ-BD; 2) Age 18-80 years; 3) Proficient in English; 4) Ability to provide informed consent. | 1) Actively psychotic or suicidal. | 2009-2015 | 2286 | Yes | Structured interview | At the start of the study |
| **UMICH** | 1) DSM-IV dx of BD-I or BD-II. | 1) Active/current substance abuse and 2) Neurological disease. | 2006-2022 | 846 | No. | Structured interview | At the start of the study. |
| **CRiB** | 1) DSM-IV dx of BD-I or BD-II; 2) Age 18-65 years; 3) On waiting list to receive psychological therapy; 4) Proficient in English; 5) Euthymic for >1 month (scoring <8 on the Hamilton Rating Scale for Depression and Young Mania Rating scale on two occasions one week apart assessing the full preceding months; 6) Ability to use a computer. | 1) Neurological illness; 2) Substance use and 3) Personality disorder. | 2016-2018 | 80 | Yes, all of the participants had to have been euthymic for at least one month, defined as scoring less than 8 on the HAM-D and YMRS | Participant-report. | At the start of the study. |
| **BARCELONA** | 1) DSM-IV dx of BD-I or BD-II; 2) Age 18-65 years; and 3) Euthymia for at least 3 months before the study enrollment defined as a score ≤8 on the Hamilton Depression Rating Scale and of ≤6 on the Young Mania Rating Scale. | 1) Intelligence quotient < 85; 2) Any medical or comorbid psychiatric condition affecting neuropsychological performance and 3) Electroconvulsive therapy within the past year. | 1998-2020 | 785 | Yes. Patients were recruited during euthymia (YMRS ≤8 and HRSD ≤8) | EHR and Informatic dataset | At the inclusion. Some variables in the database were recollected using EHR in 2019 (also lifetime treatments). |
| **DEAKIN** | 1) DSM-IV dx of BD I, BD II or BD NOS; 2) Age > 20 years; 3) Ability to provide informed consent | 1) Inability to give informed consent. | 2005-2018 | 161 | Yes. Participants were required to be euthymic on the day of assessment. | Structured interview & Self-report | At the time of assessment |
| **FACE-BD** | 1) DSM-IV dx of BD-I or BD-II; 2) Age > 16 and 3) Current affective stability = no major mood episode (mania, hypomania, mixed or depressive) during the past 4 weeks. | 1) Current mood episode (mania, hypomania, mixed or depressive) and 2) Mental retardation, dementia and related disorders. | From 2009 to 12/17/2021 | 2957 | No. | Self-report and checked by nurse/psychiatrist using medical prescription. | At the start of the study |
| **BWH/ISMSS** | 1) Age 18 to 65 years; 2) DSM-IV dx of BD-I or BD-II; 3) Current affective stability = Clinical Global Impression of Severity in Bipolar score of ≤ 3 (mildly ill) on both the Depression score and the Mania Score. | 1) History of CNS trauma, neurological disorder, ADHD or LD diagnosed in childhood; 2) Diagnosis of recent substance abuse/dependence (past 3 months); 3) Active, unstable medical problem; 4) Medications with known adverse cognitive effects (i.e., topiramate, tricyclics, anticholinergics); 5) Medications that may enhance cognition (e.g., amphetamine, dopamine agonists); 6) Benzodiazepines within 4 hours of testing and 7) ECT in the past 12 months. | 2013-2019 | 264 | Yes. | Structured clinical interview | At the start of the study |
| **MGH** | 1) DSM-IV dx of BD-I or BD-II; 2) Age 18-68 years inclusive; 3) Ability to provide informed consent; 4) Women of child bearing potential must agree to use adequate contraception; 5) Currently symptomatic, as defined as a CGI-BP-S > 3; 6) If currently taking an SGA, must be willing to either discontinue or switch to (Quetiapine) QTP and 7) Willing to be randomized to either QTP+ adjunctive personalized treatment (APT) or Lithium (Li)+APT. | 1) Unwilling or unable to comply with study requirements; 2) If maintained on thyroid medication must be euthyroid for at least 1 month before Visit 1; 3) Patients who have had intolerable side effects with QTP or Li; 4) Patients whose clinical status requires inpatient care; 5) Drug/alcohol dependence within the past 30 days; 6) Pregnant or breastfeeding; 7) History of nonresponse to Li at a serum level of ≥ 1.0 mEq/L ≥ 8 weeks and 8) History of nonresponse to QTP at doses of at least 600 mg ≥ 8 weeks. | 09/2010 - 09/2013 | 482 | No | Research data collection by study clinician | Baseline visit |
| **SYDNEY** | 1) Participants referred by General Practitioner or other mental health professional to an Australian specialist outpatient clinic with a confirmed primary diagnosis of BD-I, BD-II, SCZ-BD or BD-NOS; 2) provision of informed written consent; 3) Age 18 or over | 1) primary DSM-IV diagnosis of recurrent major depression on detailed clinical assessment. | 2/7/2006 – 11/15/2018 | 342 | No | Structured clinical interview | At the start of the study (Date of interview) |
| **UBC** | 1) DSM-IV dx of BD-I – first manic episode within the last 3 months; 2) Age 14-35 years; 3) Proficient in English and 4) Clinically stable | 1) Evidence of a previous manic episode diagnosed retrospectively on structured interview or via collateral. | 2004-2018 | 107 | No. | Baseline assessment form and/or patient's chart | At the start of the study |
| **NeuRA** | Details on inclusion criteria, participant identification and data coding are provided in Supplemental file # 2.  1) participants were from The Sax Institute’s 45 and Up Study, aged 45 years and over at baseline (baseline recruitment occurred between 2005-2009) and resident in NSW, sampled from the Services Australia Medicare enrollment database (Supplemental file #2); 2) ICD-10-AM codes in APDC/MHADC ranging from ‘F30.0’ to ‘F31.9’ for BD and/or ‘F25.0’ to ‘F25.9’ for Schizoaffective disorder, and/or >1 supply of lithium from PBS data; 3) had non-missing ethnicity information | Details are provided in Supplemental file # 2.  1) absence of relevant ICD-10-AM diagnosis codes in APDC/MHADC and/or no supply of lithium in PBS records between date range; 2) linked administrative health records that were dated posthumously (based on entries in NSW Mortality Data); 3) a gender or age mismatch between a linked administrative health data set and the 45 and Up baseline participant questionnaire. | Baseline recruitment: 2005-2009  Study Period of data inclusion: 1/1/2005-12/31/2018 | 2041 | No | Data was provided by The Sax Institute’s 45 and Up Study, NSW Government data collections, and Australian Federal Government data collections provided by Services Australia. | Longitudinally, over the study period |

**Table S1c** Variable patterns of pharmaceutical agents aggregated and averaged by region across sites (overall cohort). This data includes all subtypes of bipolar diagnosis as provided by each site.

| Pharmaceutical Agent or Class | Overall | Europe | North America | Australia * | Proportion Test |
| --- | --- | --- | --- | --- | --- |
| MSAC | 44% | 46% | 42% | 44% (41%) | < 0.01 |
| SGA | 42% | 43% | 40% | 45% (62%) | < 0.01 |
| Antidepressants | 38% | 37% | 37% | 48% (73%) | < 0.01 |
| Two or more Mood Stabilizers | 32% | 30% | 34% | 23% (56%) | < 0.01 |
| Lithium | 29% | 36% | 23% | 31% (44%) | < 0.01 |
| Lamotrigine | 22% | 20% | 24% | 17% (1%) | < 0.01 |
| Valproic Acid | 20% | 24% | 15% | 27% (32%) | < 0.01 |
| Not on any Mood Stabilizers | 18% | 10% | 25% | 23% (14%) | < 0.01 |
| Not on any medications | 18% | 5% | 21% | 1% (5%) | < 0.01 |
| Quetiapine | 17% | 16% | 16% | 26% (34%) | < 0.01 |
| Benzodiazepines | 16% | 15% | 8% | 23% (39%) | < 0.01 |
| Aripiprazole | 10% | 12% | 10% | 2% (8%) | < 0.01 |
| First Generation Antipsychotic | 10% | 24% | 1% | 2% (8%) | < 0.01 |
| Two or more SGA | 8% | 14% | 3% | 5% (28%) | < 0.01 |
| Thyroid hormones | 8% | 12% | 8% | 7% (17%) | < 0.01 |
| Olanzapine | 7% | 7% | 5% | 11% (34%) | < 0.01 |
| Stimulants/wakefulness agents | 7% | 3% | 7% | 0% (1%) | < 0.01 |
| Non-BZD sedatives | 6% | 7% | 6% | 2% (2%) | < 0.01 |
| Risperidone | 6% | 6% | 6% | 3% (11%) | < 0.01 |
| Three or more Mood Stabilizers | 6% | 4% | 7% | 7% (17%) | < 0.01 |
| Carbamazepine | 4% | 6% | 2% | 5% (5%) | < 0.01 |
| Dual Antidepressants | 3% | 3% | 4% | 4% (2%) | 0.0311 |
| Ziprasidone | 2% | 1% | 3% | 0% (2%) | < 0.01 |
| Lurasidone | 1% | 0% | 1% | 0% (2%) | < 0.01 |
| Clozapine | 1% | 1% | 1% | 0% (1%) | 0.1736 |
| Dopamine agonists | 1% | 0% | 1% | 0% (3%) | < 0.01 |
| Paliperidone | 1% | 1% | 1% | 1% (3%) | 0.6568 |
| Asenapine | 0% | 0% | 0% | 1% (1%) | 0.6051 |
| Cariprazine | 0% | 0% | 0% | 0% (0%) | NA |

MSAC= mood stabilizing anticonvulsants; Non-BZD sedatives= Non benzodiazepines sedatives; SGA=Second generation antipsychotics.

*Australian values represent cross-sectional data, excluding NeuRA, whereas the values in parenthesis include the NeuRA longitudinal cohort data. Overall values and proportion tests reflect cross-sectional data only.

**Figure S1:** Proportional meta-analysis for each individual site broken by several pharmaceutical agents or classes (a-l). All diagnoses are included.

Barcelona = University of Barcelona; BWH/ISMMS= Brigham and Women's Hospital/Icahn School of Medicine; FACE-BD = FondaMental Advanced Centers of Expertise for Bipolar Disorders; MGH = Massachusetts General Hospital; UBC= University of British Columbia; UMICH= University of Michigan; MAYO= Mayo Clinic

**
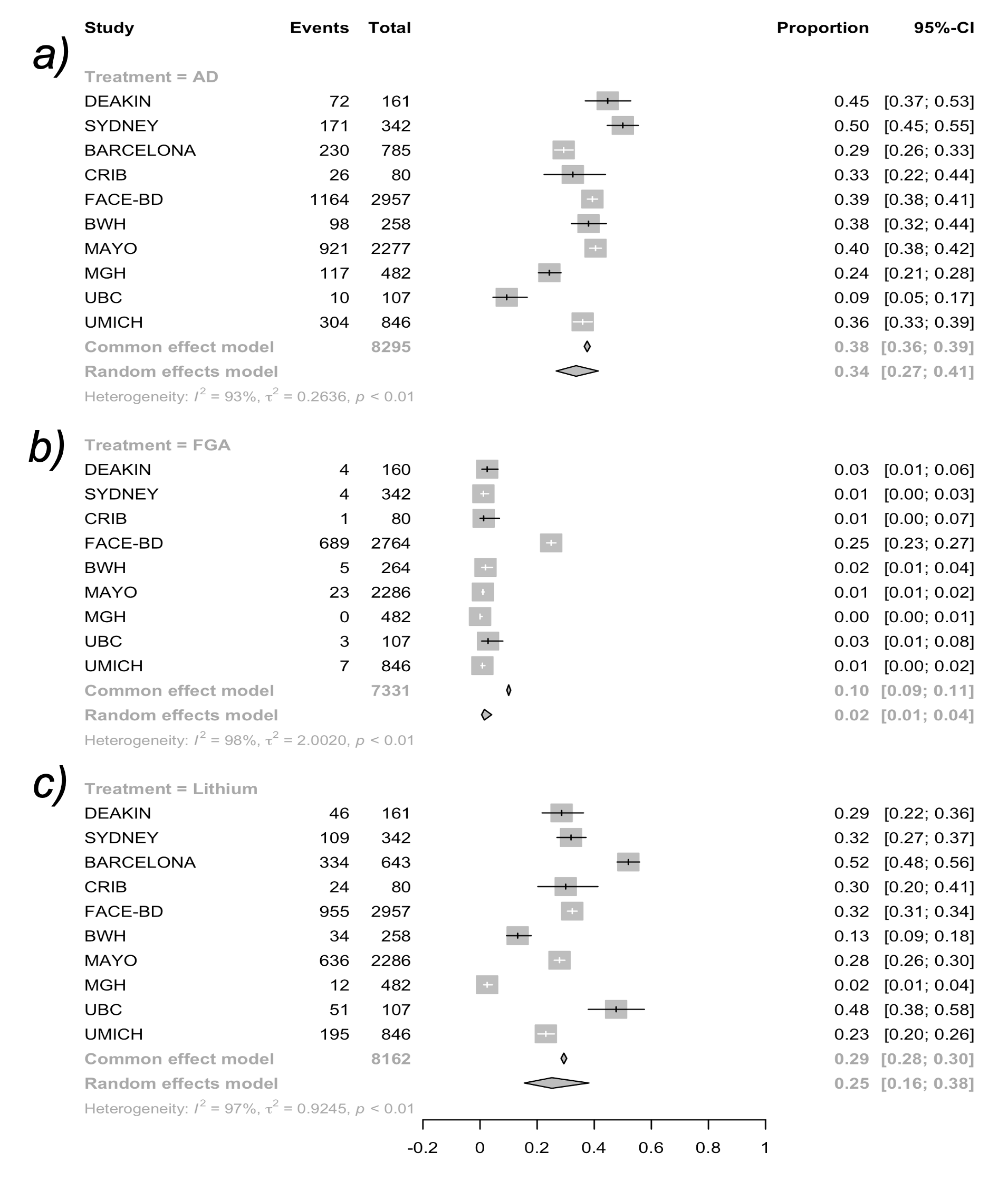
**

**
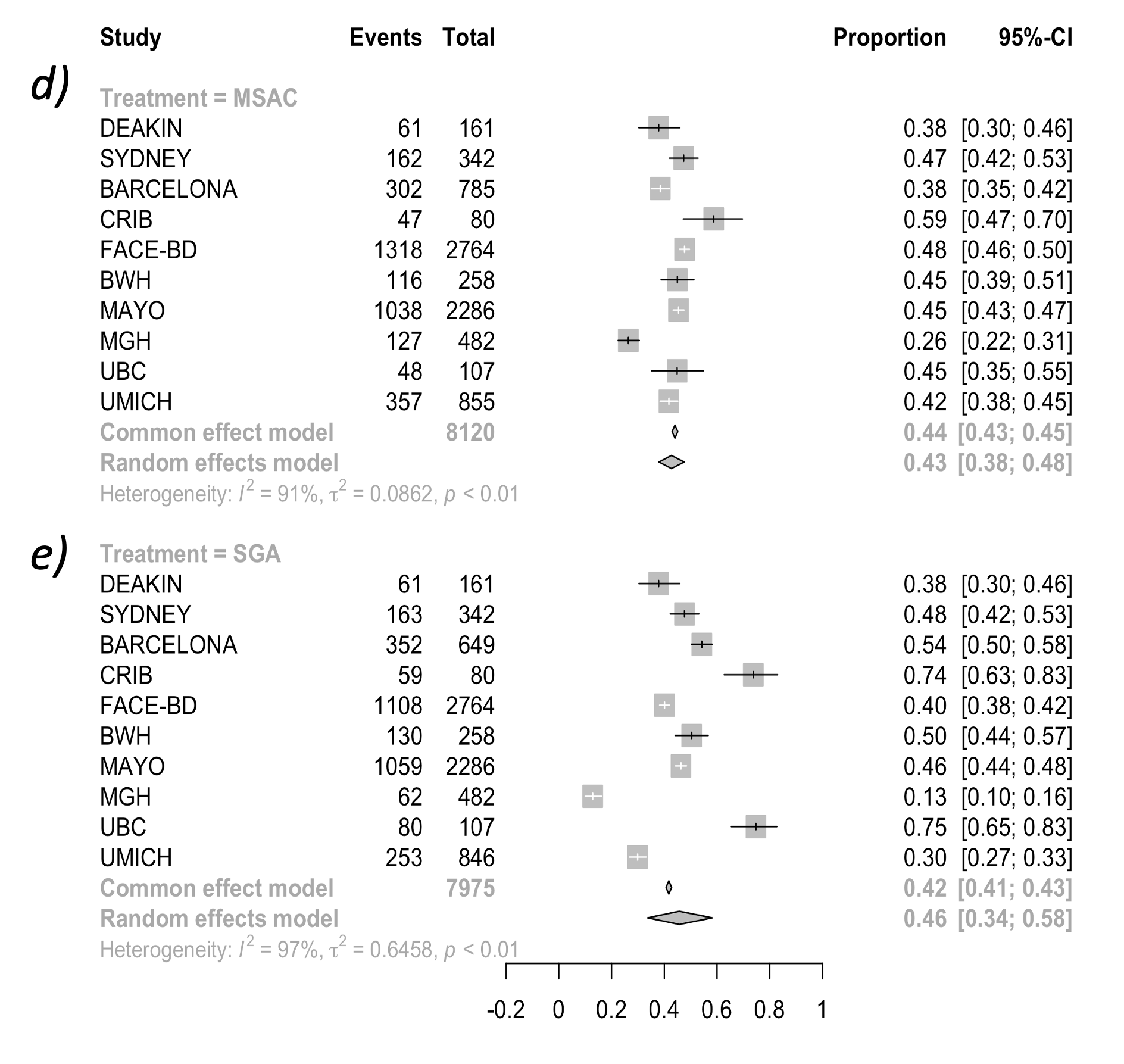
**

**
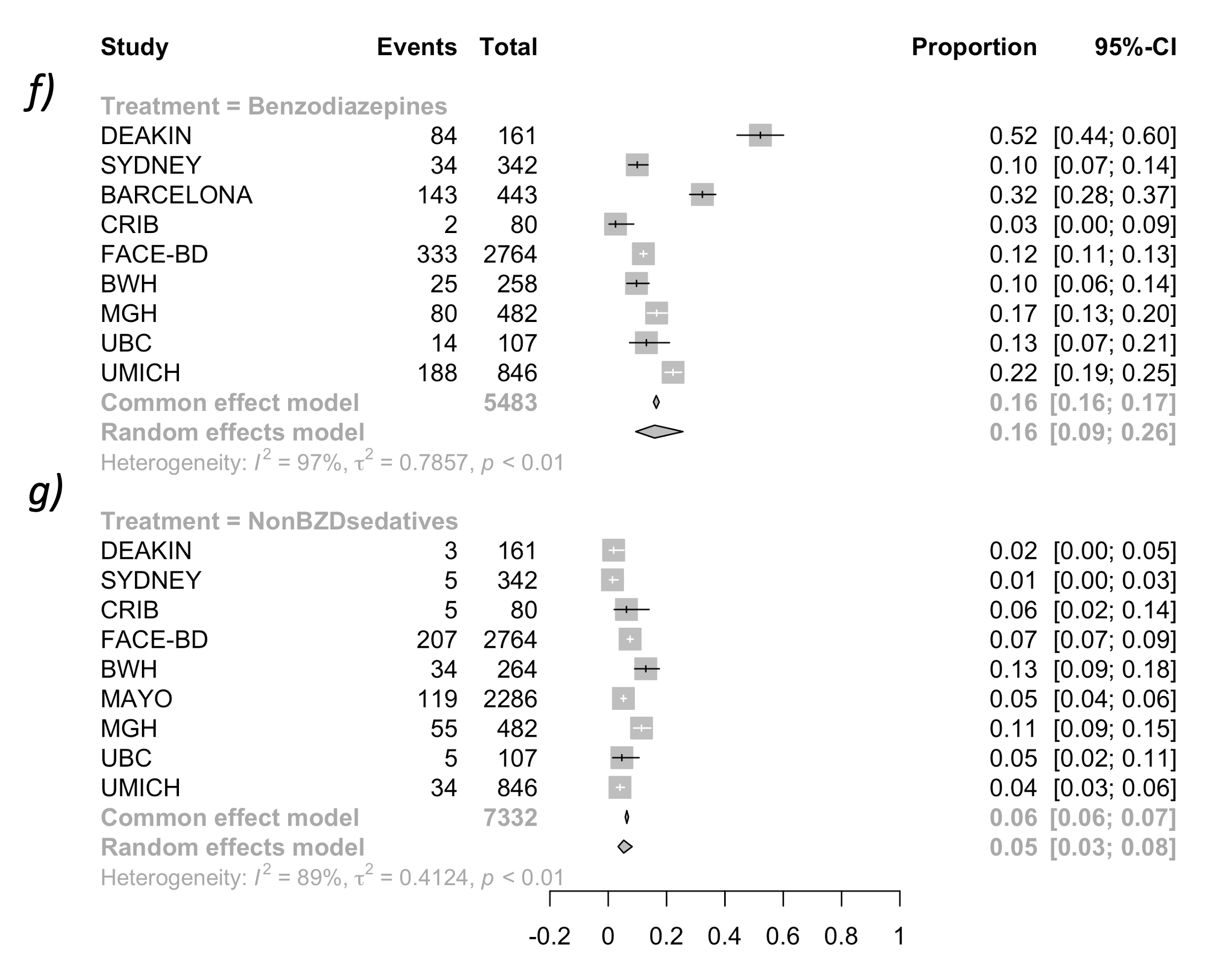
**

**
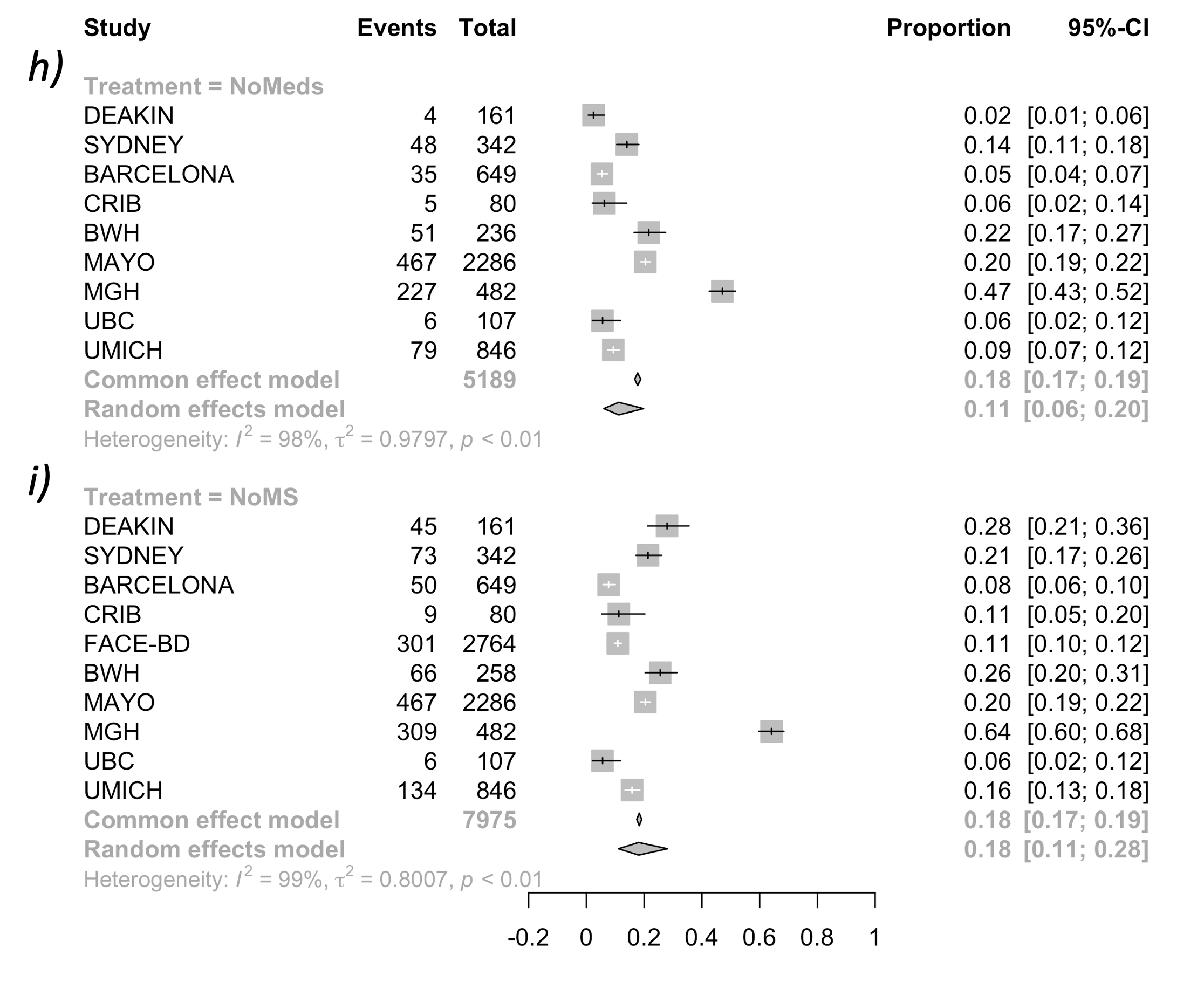
**

**
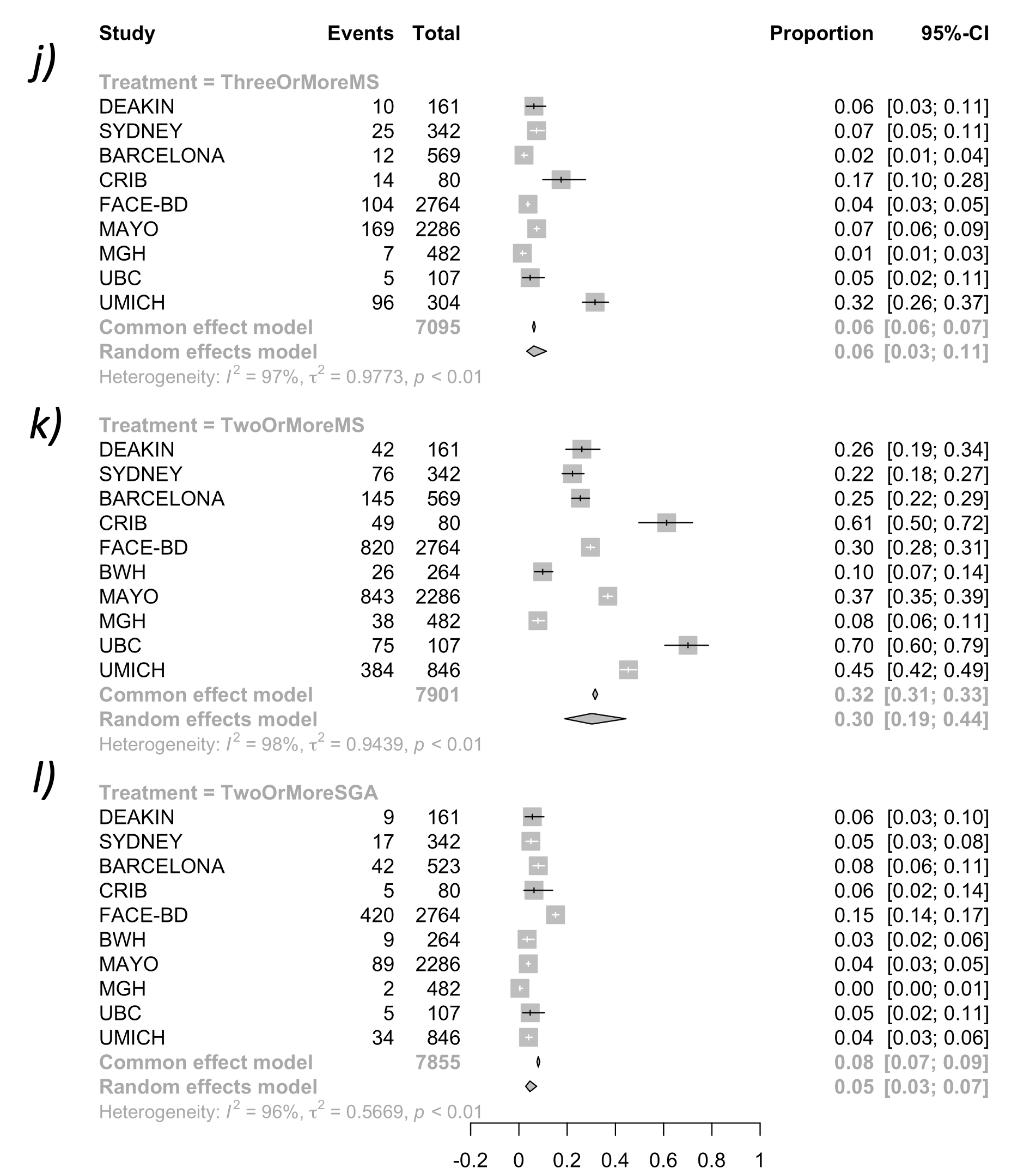
**

AD = antidepressants; FGA = first-generation antipsychotics; MS = Mood stabilizers; MSAC = mood stabilizing anticonvulsants; NonBZD sedatives = Non benzodiazepine sedatives; SGA = Second generation antipsychotics.

**Figure S2:** Subset meta-proportional analysis delineating sex, showing lithium (*a*), second-generation antipsychotics (*b*), antidepressants (*c*), and mood stabilizing anticonvulsants (*d*)

**
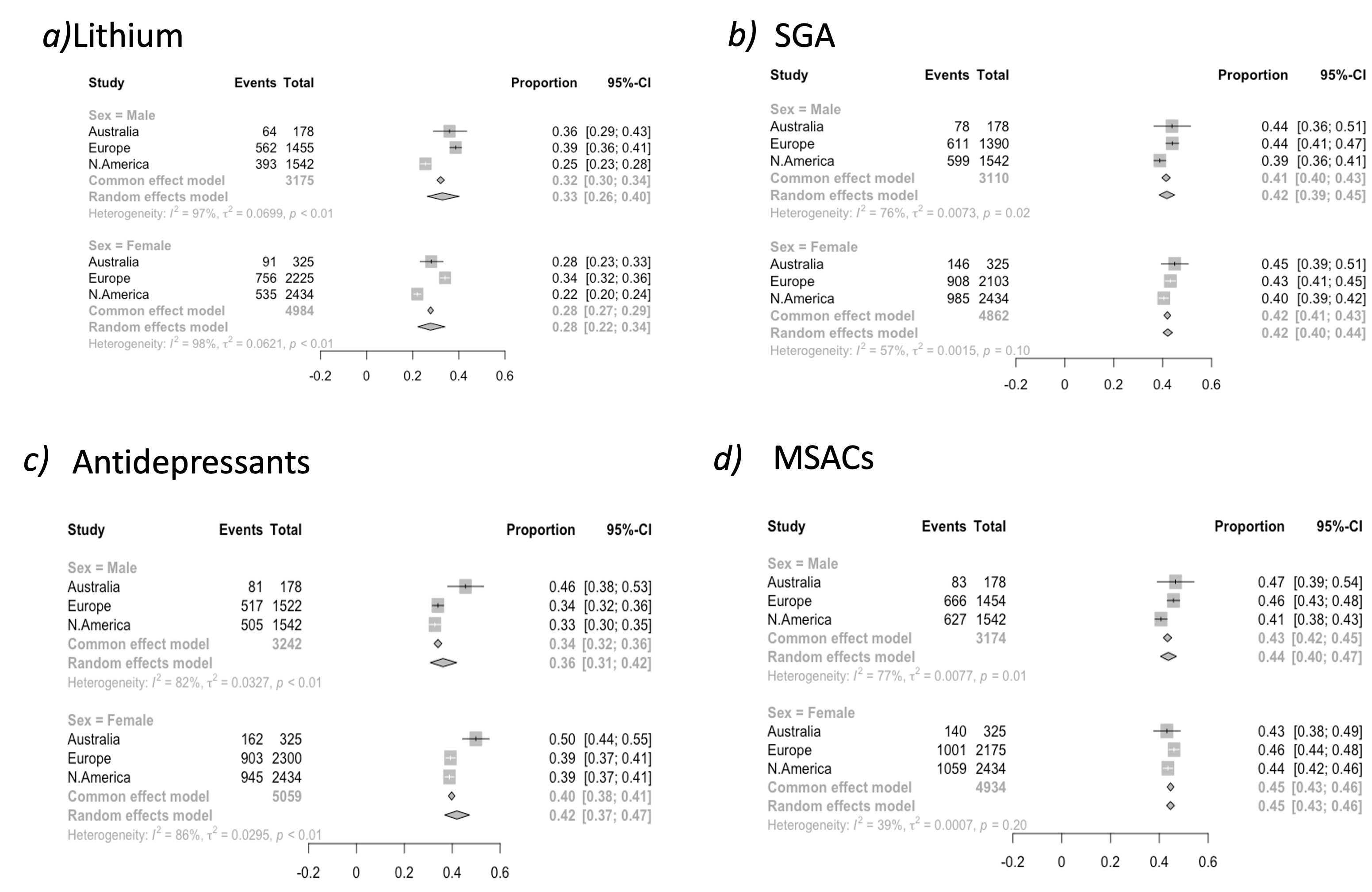
**

MSACs = mood stabilizing anticonvulsants; SGA = second-generation antipsychotics.

**Figure S3:** Proportional meta-analysis for each individual site broken by several pharmaceutical agents or classes (a-l) including data from the NeuRA cohort. All diagnoses are included.

Barcelona = University of Barcelona; BWH/ISMMS= Brigham and Women's Hospital/Icahn School of Medicine; FACE-BD = FondaMental Advanced Centers of Expertise for Bipolar Disorders; MGH = Massachusetts General Hospital; UBC= University of British Columbia; UMICH= University of Michigan; MAYO= Mayo Clinic; NeuRA = Neuroscience Research Australia

**
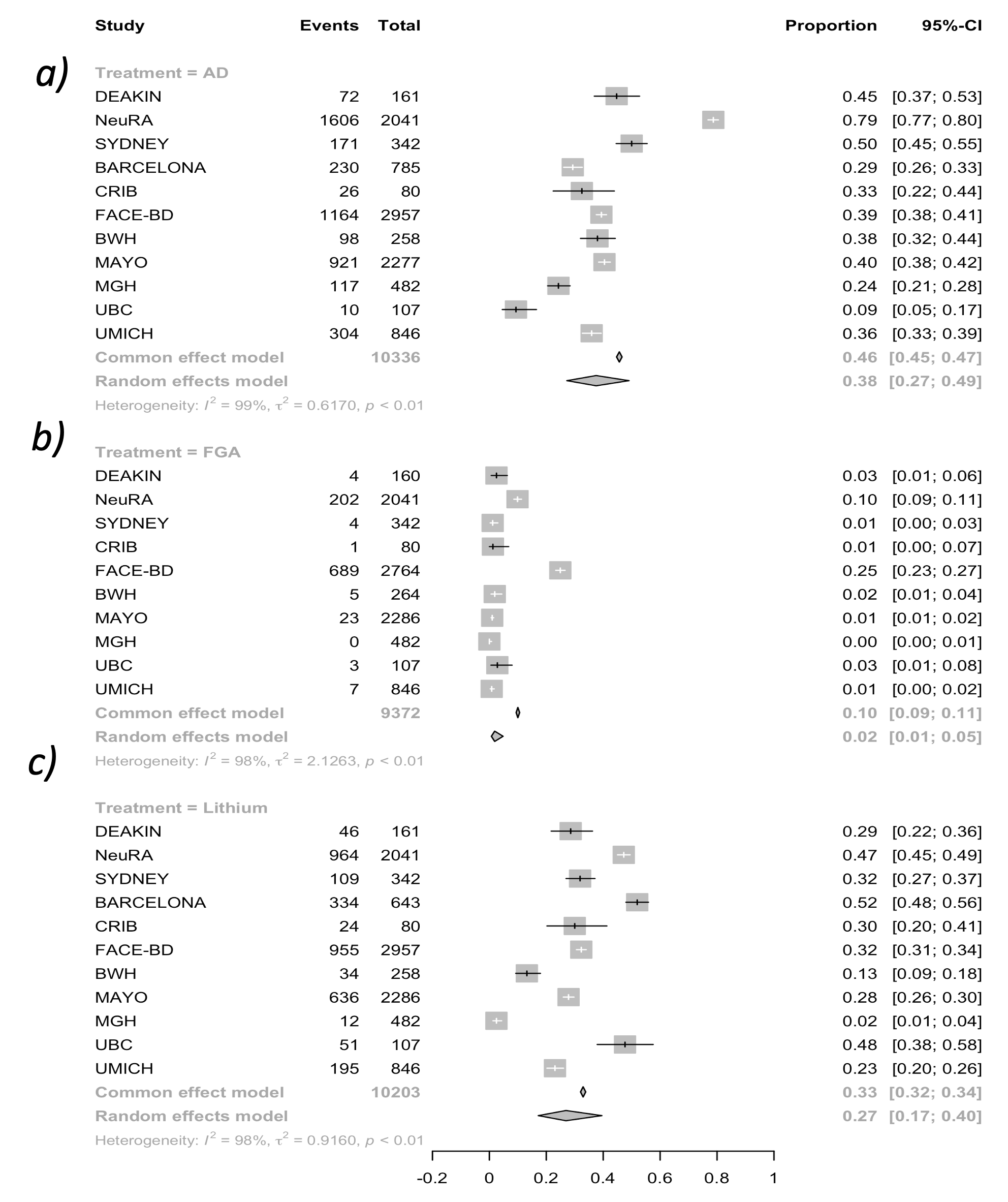
**

**
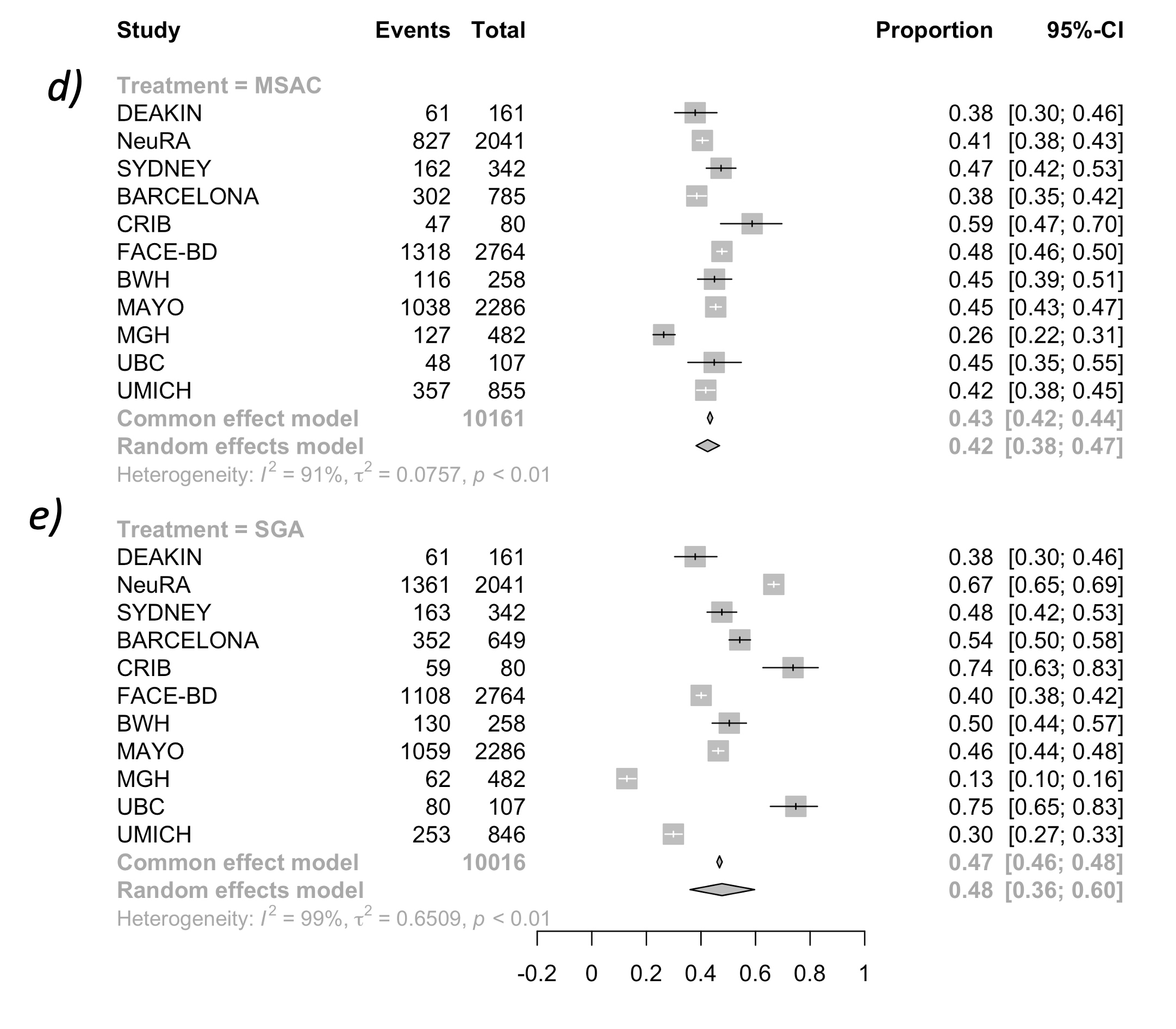
**

**
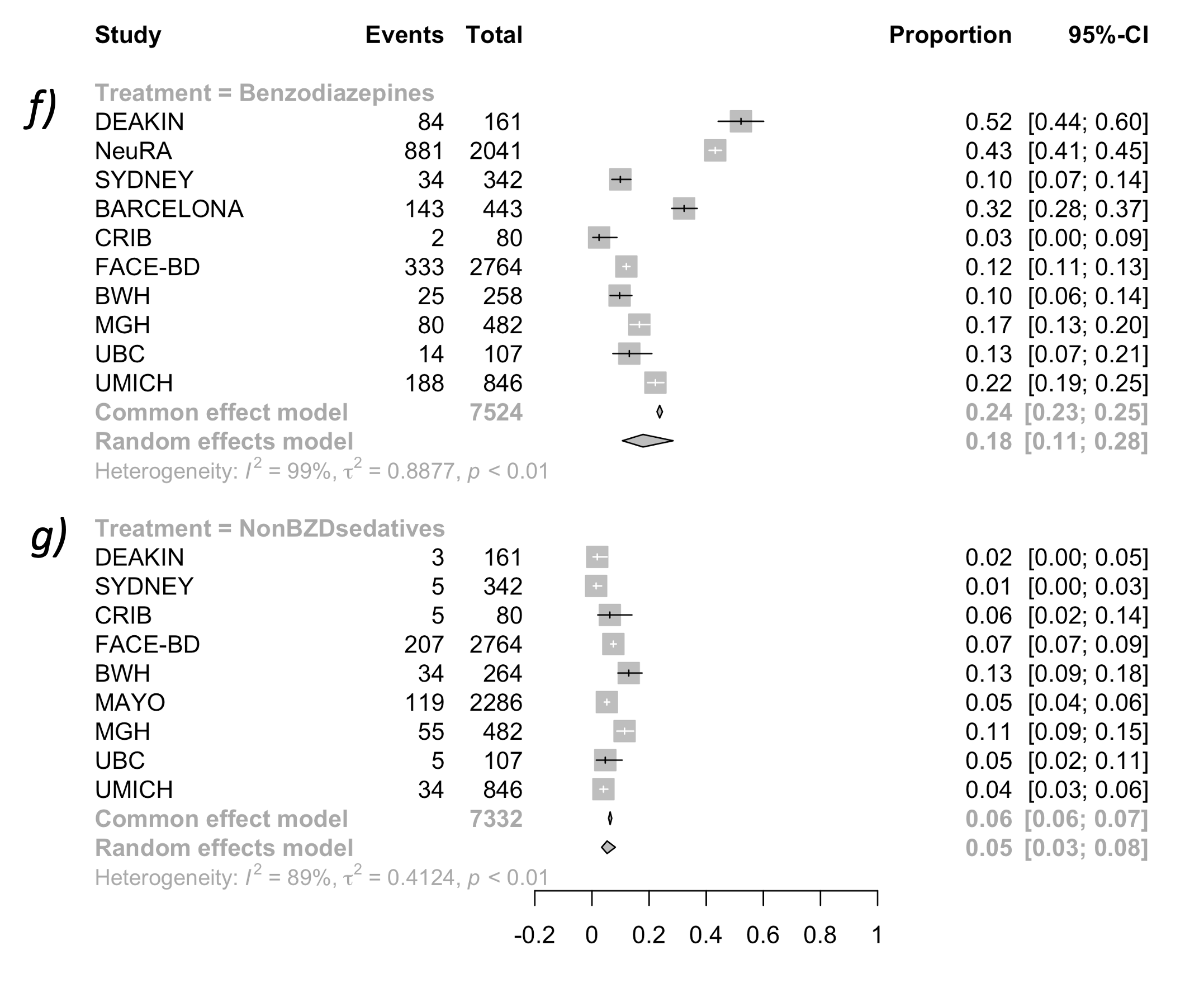
**

**
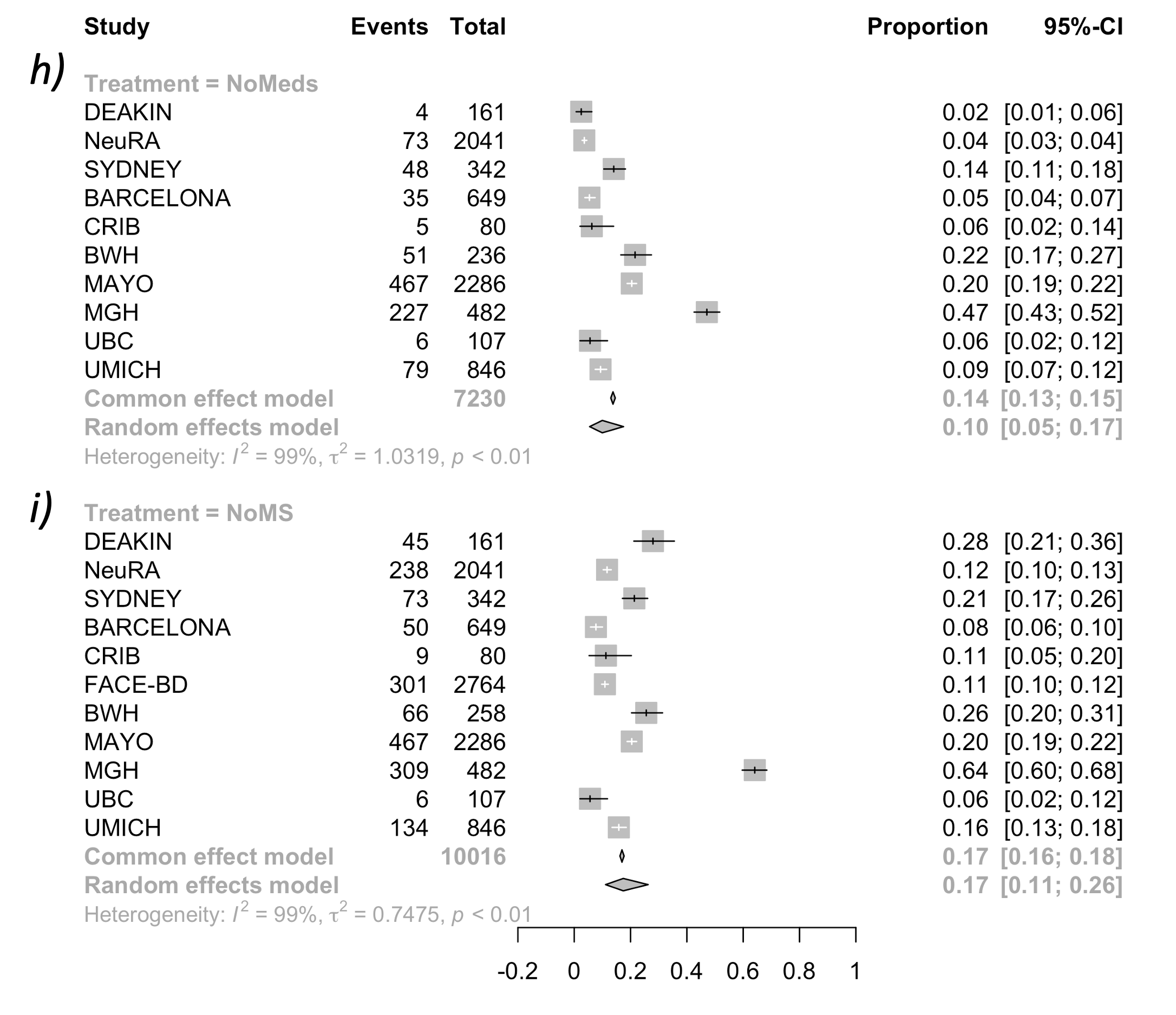
**

**
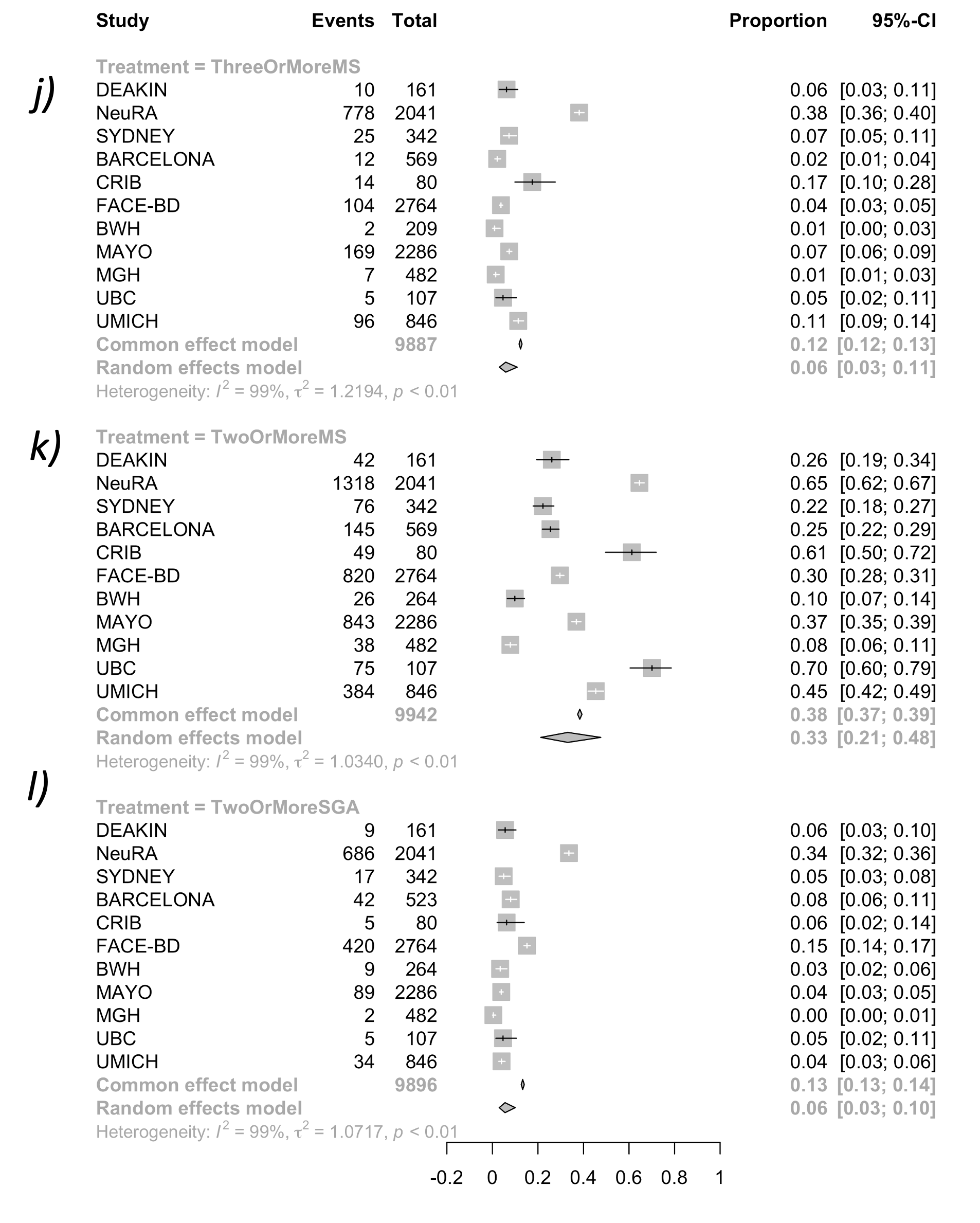
**

AD = antidepressants; FGA = first-generation antipsychotics; MS = Mood stabilizers; MSAC = mood stabilizing anticonvulsants; NonBZD sedatives = Non benzodiazepine sedatives; SGA = Second generation antipsychotics.

**Figure S4:** Proportional meta-analysis (GLMM) for antidepressants pooled by region including NeuRA cohort, stratified by antidepressants in combination with or without other psychotropics (*a*). Scatter plot with linear regression correlation between proportions with history of SUD *(b)* or comorbid anxiety diagnosis *(c)* against proportion of antidepressant use for each participating site.


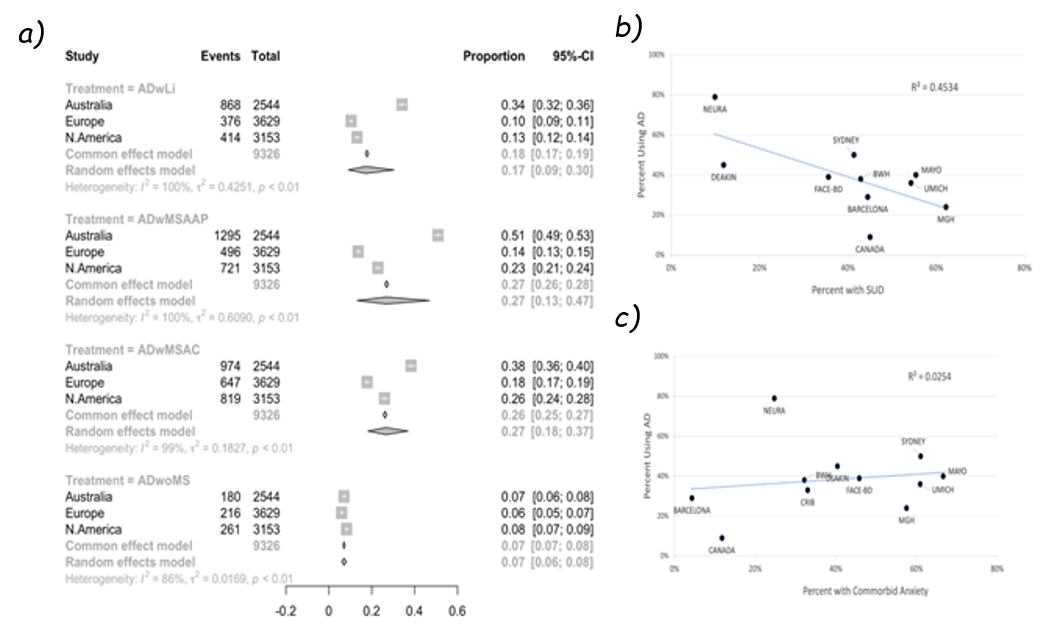


**File S2.**

**NeuRA cohort**

The NeuRA cohort sample was derived from The Sax Institute’s 45 and Up Study, which comprises 267,357 residents of the Australian state of New South Wales (NSW), who were aged 45 years and over at baseline (2005-2009), who completed a baseline questionnaire and gave signed consent for follow-up and linkage of their information to routine health databases with administrative health and claims data. This cohort is fully described elsewhere (Bleicher et al. 2022; 45 and Up Study Collaborators et al. 2008). Residents were randomly sampled from the Services Australia Medicare enrolment database of the Australian Government, which provides near complete coverage of the population. People 80+ years of age were also oversampled. Participation rate was ~19%, and participants included ~11% of the NSW population aged 45 years and over, with over sampling of rural areas. The conduct of The 45 and Up Study was approved by the University of New South Wales Human Research Ethics Committee.

Data were linked by NSW Centre for Health Record Linkage (CHeReL; http://www.cherel.org.au/) to >157 million records from NSW Government data collections, or Australian Government data collections supplied by Services Australia (formerly Department of Human Services) and were linked to self-report questionnaire data by the Sax Institute. CHeReL uses a probabilistic procedure to link records, in which records with an uncertain probability of being true matches are checked by hand, and are designed to achieve a false positive rate of ~0.5%. Deterministic linkage to Pharmaceutical Benefits Scheme (PBS) & Medicare Benefits Schedule (MBS) claims data ensures no false links. Data sources included:

1. NSW Admitted Patient Data Collection (APDC), containing records of all admitted-patient services provided by hospitals, psychiatric hospitals and multi-purpose services in the public-sector, plus private-sector hospitals and day procedure centers in NSW
2. NSW Mental Health Ambulatory Data Collection (MHADC), containing records for the care of non-admitted patients including mental health day programs, psychiatric out-patients and outreach services.
3. NSW Mortality Data, containing information on death registrations and causes of death.
4. The Pharmaceutical Benefits Scheme (PBS), containing data on the prescription and supply of all government subsidized medicines, supplied by Services Australia.
5. Medicare claims data from the Medicare Benefits Schedule (MBS), containing data on items listed on the MBS for access to professional health services accessed through general practice, private psychiatrists, clinical psychologists or allied mental health professionals, supplied by Services Australia.
6. The Sax Institute’s 45 and Up Study questionnaire (<https://www.saxinstitute.org.au/our-work/45-up-study/questionnaires/>), completed by each participant on study entry.

**Participant inclusion criteria**

Bipolar disorder cases were identified from administrative health record data, after exclusion of a small number of individuals of Aboriginal and Torres Strait Islander ethnicity, under ethical approval by the NSW Population and Health Services Research Ethics Committee (2019/ETH01615). Based on weighted evidence from the APDC, MHADC, and items listed on the PBS and MBS, individual participants were allocated into one of seven categories (Table S1). Of the 263,060 participants available for analysis, a total of N=2,149 likely bipolar cases were identified including *n*=108 individuals with missing ethnic ancestry data, who were excluded from the total sample prior to analysis. PBS records of medication supply included the period of January 1, 2005 to December 31, 2018. Relevant ICD-10-AM codes from the APDC and MHADC included:

1. Bipolar disorder ICD-10-AM codes ranging from ‘F30.0’ to ‘F31.9’
2. Schizoaffective disorder ICD-10-AM codes ranging from ‘F25.0’ to ‘F25.9’.

**Table S1.** Weighted criteria for identification of bipolar disorder (BD) cases

| **BD Case Category** | ***n* relevant ICD-10-AM Codes**  **(APDC or MHA)** | ***n* Medication supplies (PBS-listed items)** | ***n* Psychiatry or GP Mental Health Visits (MBS-listed items)** | **N BD cases** | |  |
| --- | --- | --- | --- | --- | --- | --- |
| 1 | ≥3 | ≥ 1 lithium or valproate | ≥3 | 453 | |  |
| 2 | ≥2 | ≥ 2 BD medication* | -- | 352 | |  |
| 3 | ≥2 | -- | ≥2 | 58 | |  |
| 4 | 2 | -- | -- | 41 | |  |
| 5 | 1 | -- | -- | 415 | |  |
| 6 | -- | ≥ 2 lithium† | -- | 586 | |  |
| 7 | -- | 1 lithium† | -- | 244 | |  |
| **N BD cases (Categories 1-7)** | | | | | **2,149** | |
| n BD cases with missing ethnicity (excluded) | | | | | 108 | |
| **N BD cases for inclusion** | | | | | **2,041** | |

* Bipolar Medications that were considered for Weighted Category 2 included Aripiprazole, Asenapine, Carbamazepine, Lamotrigine, Lithium, Olanzapine, Paliperidone, Quetiapine, Valproate and Ziprasidone.

† As Lithium has a single BD-specific indication, individuals with records of supply of Lithium were defined as BD cases, regardless of presence of ICD-10 code from APDC or MHA and are included in Weighted Category 6 & 7.

**Additional coding information**

1. Prior to analysis, deletion of participant data (within a collection) occurred where:
   1. a gender or age mismatch was observed between a linked administrative health data set and the 45 and Up baseline participant questionnaire, and
   2. linked administrative health records that were dated posthumously (based on entries in NSW Mortality Data).
2. Participants with ICD-10-AM records for both BD and schizoaffective disorder were included in the ‘Cohort-Data request’ table as BD cases.
3. Lifetime comorbid substance and anxiety diagnoses were determined by the presence of relevant ICD-10-AM codes in the APDC and/or MHADC data sets:
4. anxiety diagnoses ICD-10-AM codes ranged between ‘F40’ and ‘F42.9’
5. substance diagnoses ICD-10-AM codes included F10.2, F11.2, F12.2, F13.2, F14.2, F15.2, F16.2, F17.2, F18.2 and F19.2.
6. In the absence of available current depression and anxiety measures, summary data for the Kessler Psychological Distress Scale (K10) from the 45 and Up Study Baseline Questionnaire, which was completed at study entry, was employed. Total scores across the 10 items were used. Individuals with missing values for ≥2 items in this scale were excluded from analysis. For individuals with only 1 missing value, mean substitution was used for this item, based on the individuals’ responses to the remaining 9 items.
7. NSW Mortality Data revealed that 1,635 of the 2,041 analyzed BD cases (80.12%) were alive at the end of the 14-year study period.

**References**

1. 45 and Up Study Collaborators, E. Banks, S. Redman, L. Jorm, B. Armstrong, A. Bauman, J. Beard, V. Beral, J. Byles, S. Corbett, R. Cumming, M. Harris, F. Sitas, W. Smith, L. Taylor, S. Wutzke, and S. Lujic. 2008. 'Cohort profile: the 45 and up study', Int J Epidemiol, 37: 941-7.
2. Bleicher, K., R. Summerhayes, S. Baynes, M. Swarbrick, T. Navin Cristina, H. Luc, G. Dawson, A. Cowle, X. Dolja-Gore, and M. McNamara. 2022. 'Cohort Profile Update: The 45 and Up Study', Int J Epidemiol.
